# Iron Chelation Prevents Age‐Related Skeletal Muscle Sarcopenia in Klotho Gene Mutant Mice, a Genetic Model of Aging

**DOI:** 10.1002/jcsm.13678

**Published:** 2025-01-10

**Authors:** Chhanda Bose, Judit Megyesi, Oleg Karaduta, Sharda P. Singh, Sundararaman Swaminathan, Sudhir V. Shah

**Affiliations:** ^1^ Central Arkansas Veterans Healthcare System Little Rock Arkansas USA; ^2^ University of Arkansas for Medical Sciences Little Rock Arkansas USA; ^3^ Pharmacology and Neuroscience Department Texas Tech Health Sciences Center Lubbock Texas USA; ^4^ Department of Biochemistry and Molecular Biology University of Arkansas for Medical Sciences Little Rock Arkansas USA; ^5^ Internal Medicine Department Texas Tech Health Sciences Center Lubbock Texas USA

**Keywords:** catalytic iron, deferiprone, klotho, myostatin, skeletal muscle sarcopenia

## Abstract

**Background:**

A decline in skeletal muscle mass and function known as skeletal muscle sarcopenia is an inevitable consequence of aging. Sarcopenia is a major cause of decreased muscle strength, physical frailty and increased muscle fatigability, contributing significantly to an increased risk of physical disability and functional dependence among the elderly. There remains a significant need for a novel therapy that can improve sarcopenia and related problems in aging. Iron accumulation, especially catalytic iron (labile iron) through increased oxidative stress, could be one of the contributing factors to sarcopenia. Our study aimed to examine the effect of an iron chelator on age‐related sarcopenia in mice.

**Methods:**

We investigated the effect of iron chelation (deferiprone, DFP) in sarcopenia, using mice with klotho deficiency (*kl/kl*), an established mouse model for aging. Four weeks old Klotho ^−/−^ male mice were treated with 25 mg/kg body weight of iron chelator deferiprone in drinking water for 8–14 weeks (*n* = 12/group, treated and untreated). At the end of the study, gastrocnemius, quadriceps and bicep muscles were dissected and used for western blot and immunohistochemistry analysis, histopathology and iron staining. Serum total iron, catalytic iron and cytokine ELISAs were performed with established methods.

**Results:**

Treatment with DFP significantly reduced loss of muscle mass in gastrocnemius and quadriceps muscles (*p* < 0.0001). Total and catalytic iron content of serum and iron in muscles were significantly (both *p* < 0.0001) lower in the treated animals. The inhibitory factor of myogenesis, the myostatin protein in gastrocnemius muscles (*p* = 0.019) and serum (*p* = 0.003) were downregulated after 8 weeks of therapy accompanied by an increased in muscle contractile protein myosin heavy chain (~2.9 folds, *p* = 0.0004). Treatment decreased inflammation (serum IL6 and TNFα) (*p* < 0.0001, *p* = 0.005), respectively, and elevated insulin‐like growth factor levels (*p* = 0.472). This was associated with reduced DNA damage and reduced 8‐hydroxy 2 deoxyguanosine in muscle and HO‐1 protein (*p* < 0.001, *p* = 079), respectively. Significant weight loss (*p* < 0.001) and decreased water intake (*p* = 0.012) were observed in untreated mice compared to treatment group. Kaplan–Meier survival curves show the median life span of treated mice was 108 days as compared to 63 days for untreated mice (*p* = 0.0002).

**Conclusions:**

In summary, our research findings indicate that deferiprone reduced age‐related sarcopenia in the muscles of *Klotho* ^*−/−*^ mice. Our finding suggests chelation of excess iron could be an effective therapy to counter sarcopenia. However, additional studies are needed to evaluate and determine the efficacy in humans.

## 1 Introduction

Skeletal muscle is the largest organ in the body by mass, and the age associated loss of skeletal muscle mass and strength (i.e., sarcopenia) seems an unavoidable part of the aging process. There is a progressive decrease of muscle mass after about the age of 50 years at the rate of 1%–2% per year. Muscle strength also decreases by about 3% yearly after 60 years of age [1], whereas the cross‐sectional area of skeletal muscle is reduced by 25%–30% after age 70 [2]. With aging, there are decreases in the synthesis rate of muscle and mitochondrial proteins and a 20%–80% decrease in protein turnover [3]. There are several reports showing the increased ability and capacity of skeletal muscle to synthesize proteins in response to short‐term resistance exercise. However, even master‐class athletes who continue to train and compete throughout their adult lives exhibit a progressive loss of muscle mass and strength [1]. Sarcopenia is a major cause of decreased muscle strength, physical frailty and increased muscle fatigability, contributing significantly to the reduced quality of day‐to‐day life and increased risk of physical disability and functional dependence among the elderly [4, 5] (S1 and S2).

The pathologic effects of iron accumulation in tissue in iron‐overload states in various medical conditions are widely known; however, recent studies show that iron plays an important role in the pathophysiology of tissue injury even in the absence of systemic iron overload [1, 6, 7]. Critical to iron's importance in biological processes is its ability to cycle reversibly between its ferrous and ferric oxidation states. This precise property, which is essential for its functions, also makes it very dangerous because free iron can catalyse the formation of free radicals, which can damage the macromolecular components of the cell. Iron toxicity may arise from the presence of catalytic (labile) iron, found intracellularly [8, 9, 10] (S3). Increased concentration of labile/catalytic iron has been implicated in various diseases [11, 12].

Skeletal muscle represents about 40% of body mass and contains 10% to 15% of total body iron [13]. Several studies have indicated that increased iron accumulation is associated with aging in several organs including skeletal muscles and mitochondria and elevated iron load is a significant component of sarcopenia (14–16; S4–S6) [14, 15, 16]. Despite evidence for increased iron in muscle tissue and its ability to induce oxidative stress and inflammation, which have been linked to sarcopenia [17, 18, 19] (S7–S9, S10) as well as its ability to induce muscle injury and fibrosis, the role of iron in sarcopenia has not been examined previously.

Klotho, an antiaging protein, is named after the goddess who spins the threads of life. Klotho^−/−^ mouse is a laboratory animal model for human aging in general that is caused by a single gene mutation. When this gene is disrupted as in the Klotho mutant mouse (kl/kl), animals exhibit a progeria phenotype that includes shortened life span and many of the characteristic features resemble human aging (skin and muscle atrophy, sarcopenia, arteriosclerosis, osteoporosis, kidney fibrosis and infertility) [20]. It has been shown that older adults with lower plasma klotho levels have poor skeletal muscle strength [21]. Conversely, overexpression of the Klotho gene extends the life span in mice [22], and Klotho gene is known to prolong life and prevent the onset of many age‐related phenotypes [23].

We examined the effects of iron chelation in age‐related skeletal muscle sarcopenia utilizing klotho^−/−^ mice. For iron chelation, we used the most widely used oral iron chelator [24] deferiprone (1, 2‐dimethyl‐3‐hydroxypyridin‐4‐1, also known as L1). In addition to its suitability for long‐term treatment (because of oral administration), deferiprone has high‐membrane permeability as shown by its capacity to access and deplete intracellular iron pools and remove labile iron from nuclei, endosomes and mitochondria [25]. We used a dose of about one third the dose used for iron overload states and similar to the doses used in neurodegenerative disorders [10, 26].

## 2 Materials and Methods

See the Supporting Information for additional methods.

### 2.1 Animals

*Klotho* ^*+/−*^ mice were obtained from Lexicon Genetics; Mutant Mouse Regional Resource Centers, University of California at Davis, CA, and bred in Central Arkansas Veterans Health Care System Medical Unit, Little Rock, AR (VAMU) animal facility. Routine PCR using genomic DNA extracted from tail clips was performed for genotyping. Genotyping details are given in the Supporting Information methods and Figure S3. Four‐week‐old *Klotho* ^*−/−*^ male mice from the breeding colony were used for the study. Deferiprone (Sigma) 25 mg/kg body weight was administered orally with drinking water, 7 days a week for 8–18 weeks. Study design is shown in Figure S1.

### 2.2 Ethics Statement

This study was carried out in strict accordance with the recommendations of the US National Institutes of Health Guide for the Care and Use of Laboratory Animals. The protocol was approved by the Institutional Animal Care and Use Committee (IACUC approval number: 585958‐2). All efforts were made to minimize pain and suffering.

### 2.3 Biochemical Analysis

Blood samples were collected for determination of hematological and biochemical parameters at sacrifice. All biochemical parameters were measured using VetScan (Abaxis Veterinary Diagnostics, Union City, CA) at VAMU. For hemoglobin measurements, during the study, blood was collected biweekly by retro‐orbital approach under isoflurane anesthesia. Hemoglobin and hematocrit were determined with HEMAVET950 FS (Drew Scientific, Inc., Waterbury, CT).

### 2.4 Muscle Iron Staining and Assessments of Muscle Pathology

Section (6–8 μm) from fixed, paraffin‐embedded gastrocnemius muscles was stained with Pearls Prussian blue and haematoxylin–eosin (H&E) stains. The presence of centrally nucleated muscle fibres, muscle atrophy and pathological changes were examined with H&E staining. All histology and immunohistochemistry studies were conducted at the University of Arkansas for Medical Sciences by a trained pathologist. Muscle samples were processed for H&E, Perl's Prussian blue and immunohistochemical staining as described before (S12). In brief, sections were stained for iron deposition using Perl's Prussian blue kit (Polyscience, Warrington, PA) according to the manufacturer's instructions. After deparaffinization, muscle sections were incubated in equal amounts of 4% potassium ferrocyanide and 4% HCl solution for 20 min. After incubation, slides were washed twice with 1X PBS and counterstained with Nuclear Fast Red solution (Sigma‐Aldrich, Saint Louis, MO) for 30 min. A qualified pathologist (JM) with extensive expertise in a blinded fashion captured all images for histopathology and analysis using a Nikon Eclipse E800 microscope with a Cool SNAP camera (Nikon Metrology, Inc., Brighton, MI).

### 2.5 Bleomycin‐Detectable Iron Assay for Catalytic Iron

At the end of the study, catalytic iron (capable of catalysing free‐radical reactions) in the serum samples of untreated and treated mice was measured by the bleomycin assay as described earlier (S13) as follows: 1 mL of the reaction mixture contained in order: 0.5 mL of calf thymus DNA (1 mg/mL), 0.05 or 0.1 mL of bleomycin sulphate (1 mg/mL), 0.1 mL of MgCl2 (50 mM), 0.1 mL of sample, 0.1 mL of Chelex treated pyrogen‐free water, 0.1 mL of ascorbic acid (8 mM) and either 0.05 mL of HCl (10 mM) or 0.1 mL of imidazole (1.0 M, pH 7.3) to adjust to pH 7.4. Sample blanks were identical except that bleomycin was omitted. The samples were then incubated at 37°C for 2 h with shaking. The reaction was stopped by adding 0.1 M EDTA mixed with 1 mL of thiobarbituric acid (1% wt/vol in 50 mM NaOH) and 1 mL of 25% HCl (vol/vol). The reaction mixture was then heated at 100°C for 15 min and cooled, and the resulting chromogen was measured using a spectrophotometer by its absorbance at 532 nm. A standard curve was prepared using known amounts of FeCl3 in Chelex‐treated pyrogen‐free water. The amount of bleomycin‐detectable iron in the test sample was calculated from the standard curve, and the results were expressed as nmol/mg of serum protein. Protein was measured using a Bio‐Rad Laboratories, Inc. (Hercules, CA) reagent. All reagents except for the sample to test were prepared in Chelex‐treated pyrogen‐free water and shaken with Chelex‐100 to remove as much contaminating iron as possible.

### 2.6 Immunohistochemistry

Immunohistochemistry staining was performed on 6‐ to 8‐μm muscle sections. Microwave antigen retrieval was performed by using Antigen Unmasking Solution (Vector's Lab). Myosin heavy chain, myostatin and 8‐OHdG were detected by using appropriate antibodies (details of the antibodies are given in Table S1). A goat antimouse, goat antirabbit, secondary antibody linked to horseradish peroxidase (Dako North America, Inc., Carpentaria, CA) was used with a peroxidase substrate kit (AEC, Vector Laboratories, Burlingame, CA). Slides were counterstained with haematoxylin. Three different mice from each group were used. Three random images from each section were taken, and quantitative analysis was performed by a trained pathologist (JM) blinded for the study, using a digital camera (Nikon Digital Sight DS‐Ri1, Nikon Corporation, Japan) attached to a NIKON Eclipse 800 microscope at 440X magnification. The number of positively stained cells/mm^2^ was determined using NIS‐Elements Analysis software (Nikon).

### 2.7 Western Bot Analysis

Western blots were done as before cell extracts were analysed by SDS‐PAGE utilizing 4%–20% Tris‐glycine separating gel (Invitrogen Life Technologies; Carlsbad, CA). Equal amounts of protein were loaded into each lane, and the fractionated protein was electro‐blotted onto nitrocellulose membranes at 30 V for 1 h at room temperature. Membranes were blocked in 1X clear milk‐blocking buffer milk (Pierce; Rockford, IL) for 1 h at room temperature and then probed with primary antibodies, antimyostatin (GDF8), antimyosin heavy chain, HO‐1 and MyoD (antibody details in Table S1), diluted to 1:1000 in 5% nonfat dry milk in 1XTBST and incubated for overnight at 4°C using gentle shaking. Membranes were washed five times (5 min each) with Tris‐buffered saline‐Tween 20 [TBST; 20 mM Tris HCl (pH 7.6), 137 mM NaCl and 0.2% (vol/vol) Tween 20] and incubated with horseradish peroxidase‐coupled anti‐IgG (secondary antibody, dilution 1:2000) for 1 h, at room temperature. Enhanced Chemiluminescence (SuperSignal West Pico Chemiluminescent Substrate, Thermo Scientific, IL, USA) and fluorescence detection steps were followed per the manufacturers' instructions for visualization of the bands. For the loading control and normalization of the protein, at the end of the experiments, nitrocellulose membranes were stripped with restore western blot stripping buffer (ThermoFisher Scientific) and reprobed with HRP conjugated GAPDH (S12, S14).

### 2.8 Assessment of Oxidative Damage

Oxidative biomarker, 8‐hydroxy‐2 ‐deoxy guanosine (8‐OHdG) was examined by immunohistochemistry. For quantitative analysis of immunohistochemical staining, muscle sections from three different mice from each group were used. The number of positively stained cells/mm^2^ was determined using NIS‐Elements Analysis software (Nikon) at 40X magnification. Quantitative analysis was performed by a trained pathologist (JM) blinded for the study. Heme oxygenase (HO‐1) was examined in muscle samples by western blot analysis, utilizing appropriate antibodies as described in Table S1.

### 2.9 TUNEL Assay for Detection of DNA Damage

A terminal deoxynucleotidyl transferase dUTP‐mediated nick‐end labelling (TUNEL) assay was utilized to assess and validate apoptotic cell death. TUNEL staining was performed using an in situ cell death detection kit (Roche Diagnostics Corp., Indianapolis, IN), following the manufacturer's protocol in the DNA Damage Core Facility, UAMS.

### 2.10 Lifespan Study

Mice were monitored and recorded for survival rates up to 18 weeks after treatment. Kaplan–Meier analysis (log‐rank test) was performed to compare the survival in both groups.

### 2.11 Statistical Analysis

All statistical analysis was performed using Graph Prism Pad version 10. The significance of differences among groups for all parameters was evaluated by one‐way analysis of variance (ANOVA), followed by Tukey's post hoc test or by the unpaired two‐tailed Student's *t* test, when only two groups were compared. Differences were considered significant at a two‐tailed *p* < 0.05. Statistical tests (*p* values) used for each experiment are mentioned in the figure legends. Data presented here are expressed as means ± SD.

## 3 Results

### 3.1 Change in Intracellular Iron Homeostasis With DFP Treatment

**FIGURE 1:**
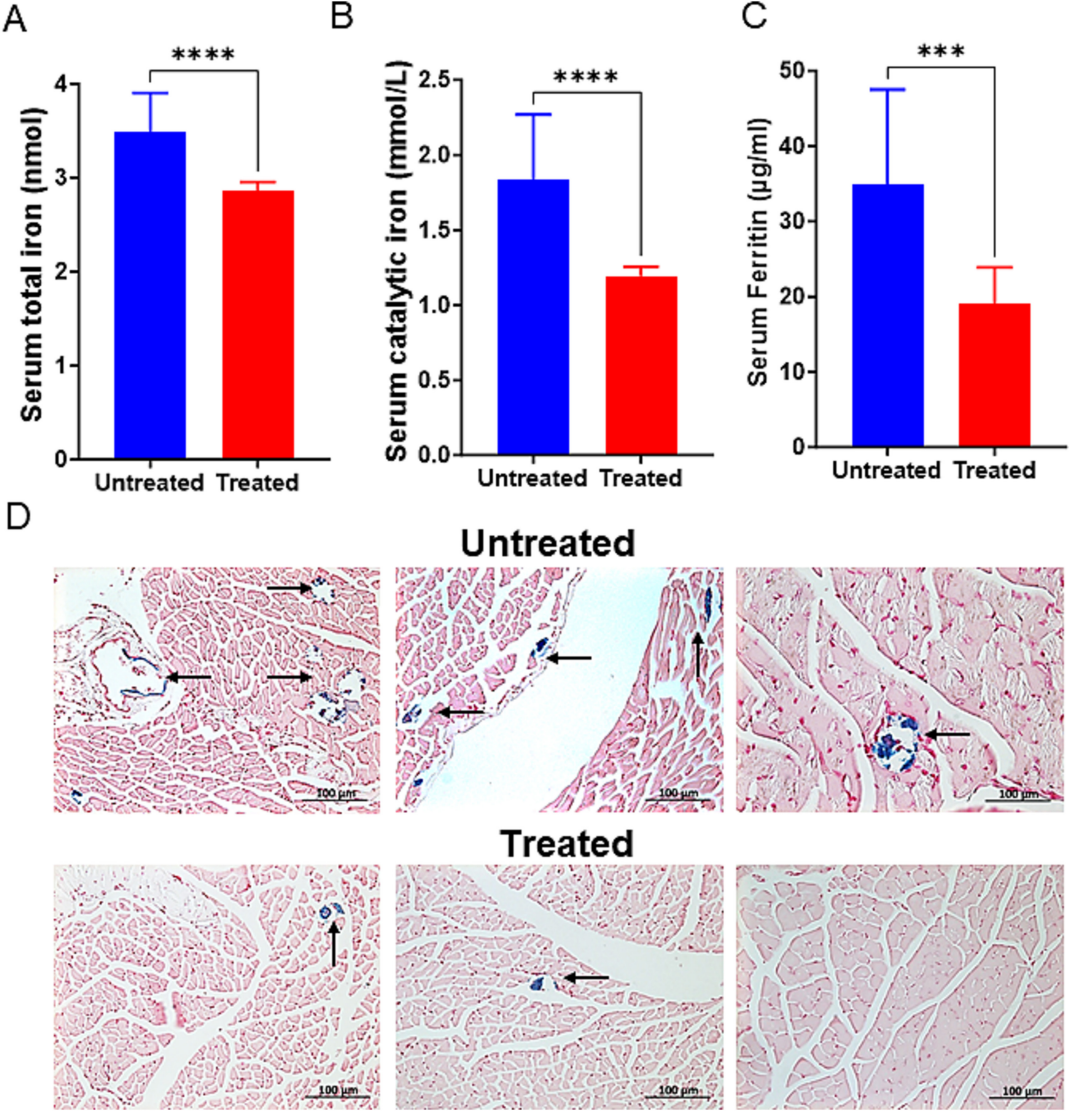
DFP changes the catalytic iron and iron accumulation in serum and muscles of Kl^−/−^ mice. Histograms show the data of the serum total iron (A) and (B) bleomycin‐detectable iron (catalytic iron) and (C) ferritin levels for, Kl^−/−^ control and treated with 25 mg/kg deferiprone for 8 weeks (*n* = 12 each group). ***p = 0.0006, ****p < 0.0001, as analysed by two tailed, Student's unpaired *t* test. (D) bright‐field image shows Prussian Blue positive staining (arrows) for presence of iron (blue) in gastrocnemius muscles from untreated and treated groups. More iron accumulation was seen in untreated mice than treatment group. Sections were counterstained with Nuclear Fast Red (*n* = 3, representative images of each mouse from both groups are shown). All images are at X20.

Iron, which is an essential nutrient, can be harmful when in excess [27], by causing increased oxidative stress in cells, [8, 10, 12] and in skeletal muscle [27, 28, 29]. We measured total iron, catalytic iron and nonheme iron ferritin, in serum and gastrocnemius muscles. The total and catalytic iron content of serum were significantly greater in the untreated group (3.48 ± 0.41 nmol and 1.84 ± 0.43 μmol/L nmol) than in the treatment group (2.86 ± 0.09 nmol and 1.23 ± 0.06 μmol/L), total and catalytic iron, respectively, (Figure 1A,B, *p* < 0.0001). Ferritin level was ~1.8‐fold higher in the untreated mice serum compared to the treatment group, 34.90 ± 12.65 and 19.75 ± 4.74 μg/mL, respectively (Figure 1C; *p* = 0.0006). To examine the iron content in tissues, gastrocnemius muscle cross‐sections were stained for free iron. The content of free iron was elevated in Klotho^−/−^ and wild type old mice as shown by Prussian blue staining in muscles from untreated animals compared to the treated group (Figures 1D and S7).

### 3.2 Effect of Deferiprone on Muscle and Body Weights, Water Consumption and Survival

**FIGURE 2:**
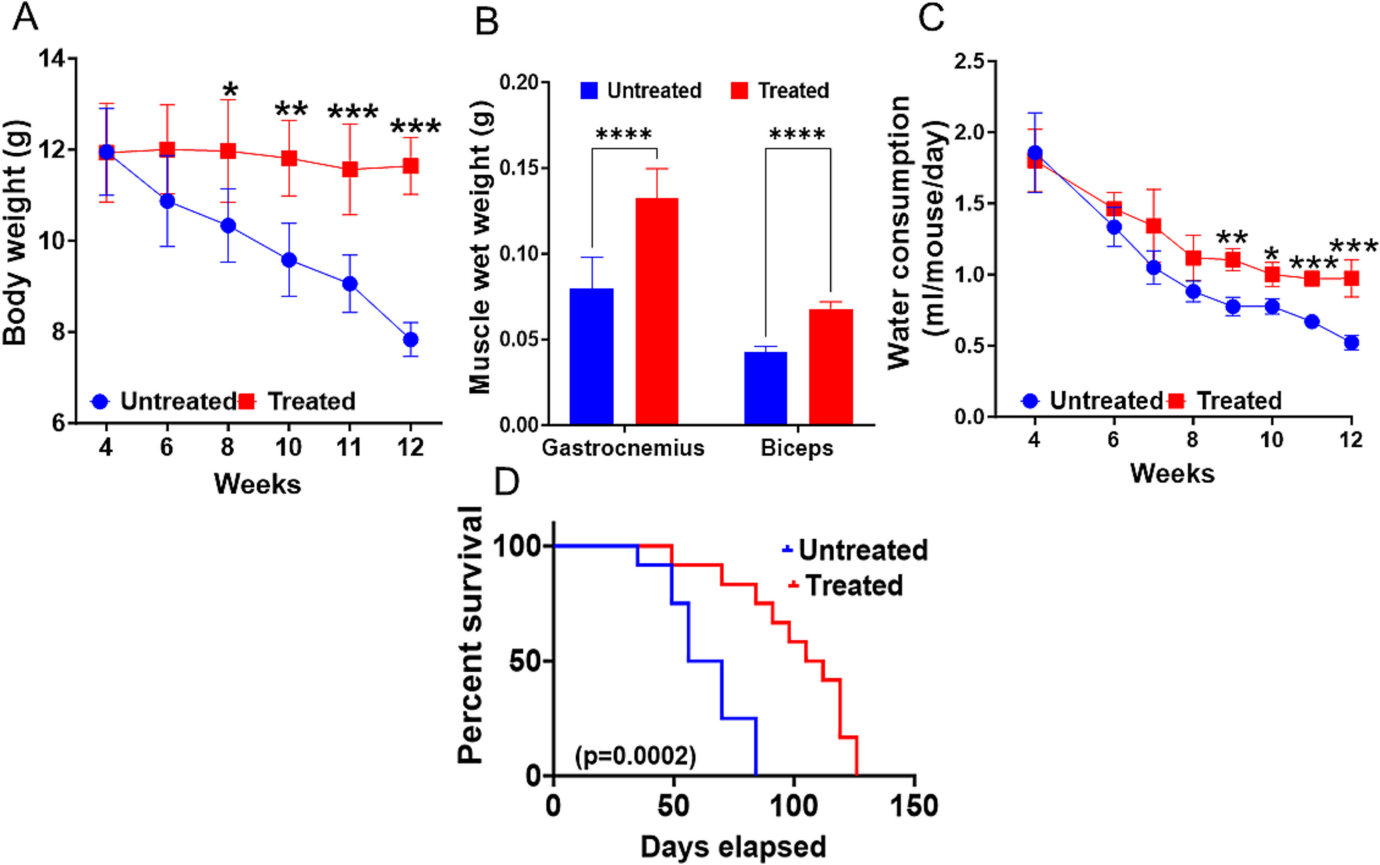
DFP treatment attenuated the muscle weights and life span of Kl^−/−^ mice: (A) weekly body weights for untreated and treated mice. Gradual body weight loss was observed in untreated mice. (B) Skeletal muscle (gastrocnemius and biceps) weight (wet weight) of untreated and treated mice at 12 weeks. Asterisks are significant changes relative to the control group as assessed by a two‐tailed, unpaired Student's *t* test. (C) Weekly water intake by both groups of mice. Significant differences between groups as calculated with a two‐tailed, unpaired *t* test with Welch correction *p < 0.05, **p < 0.01, ***p < 0.001 for body weight and water intake (*n* = 12 each group). (D) Kaplan–Meier survival curve (log‐rank test) for klotho^−/−^ mice untreated and treated with deferiprone. Treated mice were significantly long‐lived (*n* = 12 each group).

At the beginning of the study, body weights were similar (11.9 ± 0.9 g) in both groups. As shown in Figure 2A, the body weights of untreated mice gradually declined from 5 weeks of age. Significant weight loss was noted from 9 weeks (from 10.00 ± 0.88 g, to ~7.8 ± 0.0.37 g, *p* < 0.05–0.0001), and total body weight in untreated groups reduced by ~34.4% by 12 weeks compared to the mice treated with DFP 11.87 ± 1.16 g to 11.63 ± 0.62. Figure 2B presents the average skeletal muscle weights from both groups of mice at the end of the therapy. The weights of biceps and gastrocnemius muscles were significantly different (*p* = 0.0006, *p* < 0.0001), 0.043 ± 0.003 versus 0.067 ± 0.004 g of biceps and 0.082 ± 0.018 versus 0.132 ± 0.017 g of gastrocnemius in untreated and DFP treated groups, respectively. DFP was given in drinking water with free access to water; Figure 2C shows the average intake of water by both groups per week. As illustrated, the rate of water consumption started decreasing from ~9 weeks of age and decreased gradually; by 12 weeks, it was significantly less than DFP treated group (*p* < 0001, Figure 2C).

### 3.3 Life span

**FIGURE 3:**
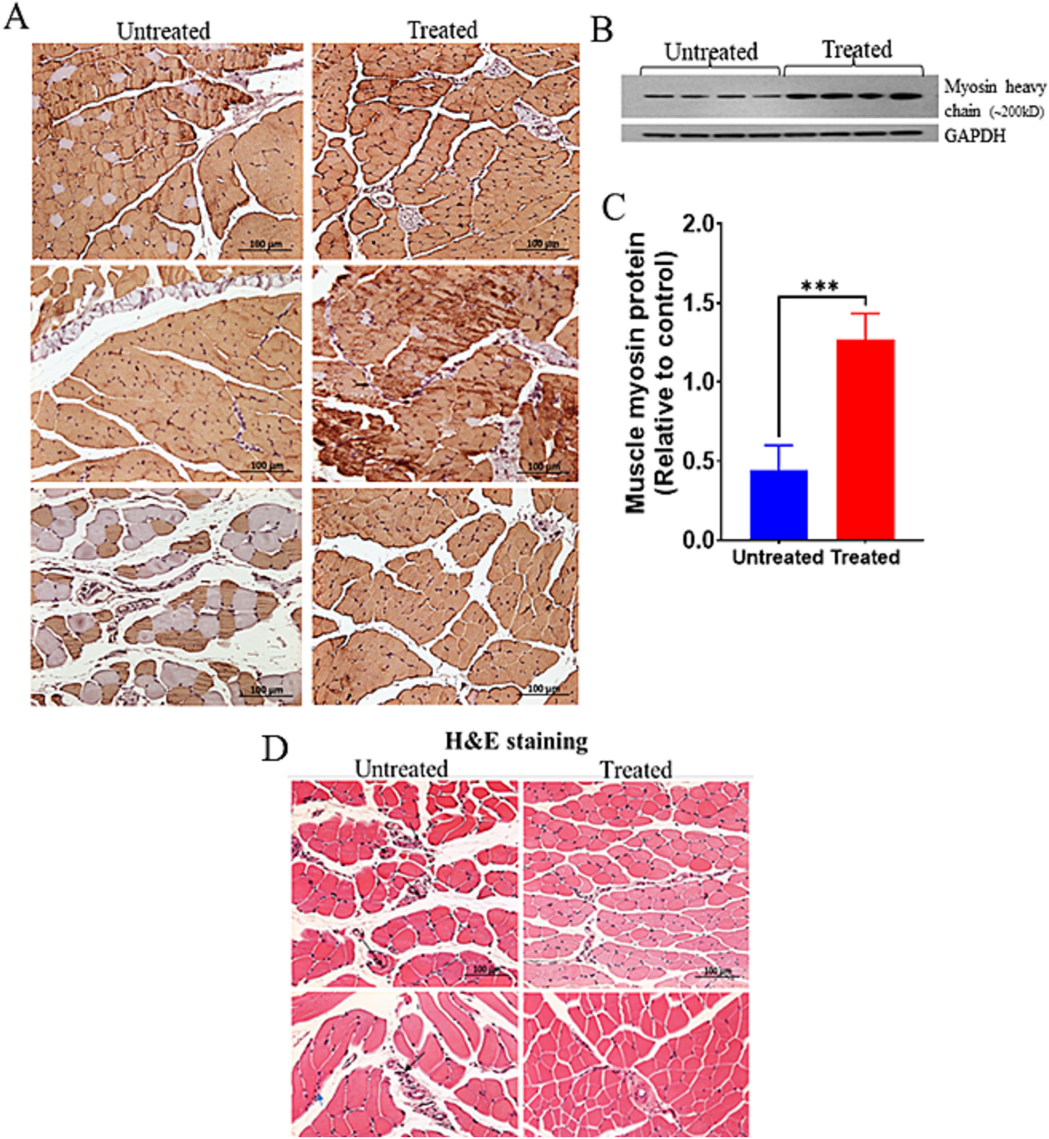
The major contractile protein myosin heavy chain is upregulated with iron chelation: (A) representative IHC images of myosin heavy chain (*n* = 3 per group, representative images of each mouse from both groups are shown) and western blots for myosin. (B) Analyses on gastrocnemius muscles from untreated and treated with DFP. All images at X20. Panel (C) shows the quantitative analyses of western blots (*n* = 4 each group). Protein bands were normalized with GAPDH. Results are presented as expressions relative to control. Data are shown as mean ± SD. ***p = 0.0004 denotes levels of significant differences between groups as calculated with a two‐tailed, unpaired Student's *t* test. (D) H&E staining of sections from gastrocnemius muscles. Representative images (*n* = 2 each group) demonstrate the characteristic pathological changes in gastrocnemius muscle samples from untreated klotho^−/−^ mice, such as atrophy (arrows) and infiltration of inflammatory cells (arrow heads). All images 400X magnification. Scale bars set at 100 μm for all.

It has been reported that *Klotho* ^*−/−*^ mice have a shorter life span. We examined whether iron chelation extends the lifespan of *Klotho* ^*−/−*^ mice. Treated and untreated mice were monitored and recorded for survival rate (*n* = 12 per group). DFP‐treated mice maintained a survival time significantly higher than untreated mice; Figure 3D shows the Kaplan–Meier survival curves. In the untreated group, the age range of mortality was 8 to 14 weeks, and 50% mortality reached at ~8 weeks. In the treated group, the age range of mortality was 12–18 weeks with 50% mortality at weeks ~14. Moreover, the median survival of the treated mice significantly increased by 108.5 days relative to untreated control mice, which was 63 days (log‐rank test, *p* = 0.0002, Kaplan–Meyer life span analysis and log‐rank test) (Figure 2D), resulting in significant prolongation of the life span by ~28 days in the treatment group.

### 3.4 Effects of DFP on Skeletal Muscle Histopathology and Muscle Myosin (MyCH)

Histological examination of gastrocnemius muscles using H&E staining revealed more muscular dystrophy at 12 weeks of age characterized by myonecrosis, central nucleation and small atrophic fibres (Figures 3D and S4) in untreated mice than treatment group of mice. An increased number of muscle cells with centrally located nuclei were seen in untreated muscle sections. No significant increase in central nucleation was observed in DFP‐treated mouse muscles (Figure S4). These data indicate ongoing muscle degeneration in untreated mice compared to treated mice. Moreover, infiltration of mononuclear cells was observed in the untreated skeletal muscle with smaller disruption of the basal lamina, as observed by IHC of muscle sections from both groups (Figures 3D, S4 and S5). Further, we examined the expression of the myogenic differentiation marker, MyHC, which is the major structural protein in myotubes. As shown in Figure 3A, the DFP‐treated mice muscles exhibited an increased expression of MyHC compared with the untreated mice. IHC staining of gastrocnemius muscle cross‐sections revealed that untreated muscles have less reactivity for MyCH compared to the treated group (Figure 3A). Similar results were observed in wild type old mice (Figure S8A). IHC findings were further confirmed by western blot analysis, as Figure 3B shows there was a decreased level of MyCH protein in untreated group, and DFP treatment elevated the protein levels by ~2.9‐fold in gastrocnemius muscle (*p* = 0.0004, Figure 3C).

### 3.5 Inhibition of Skeletal Muscle Myostatin With Altered IGF1 With DFP Treatment

**FIGURE 4:**
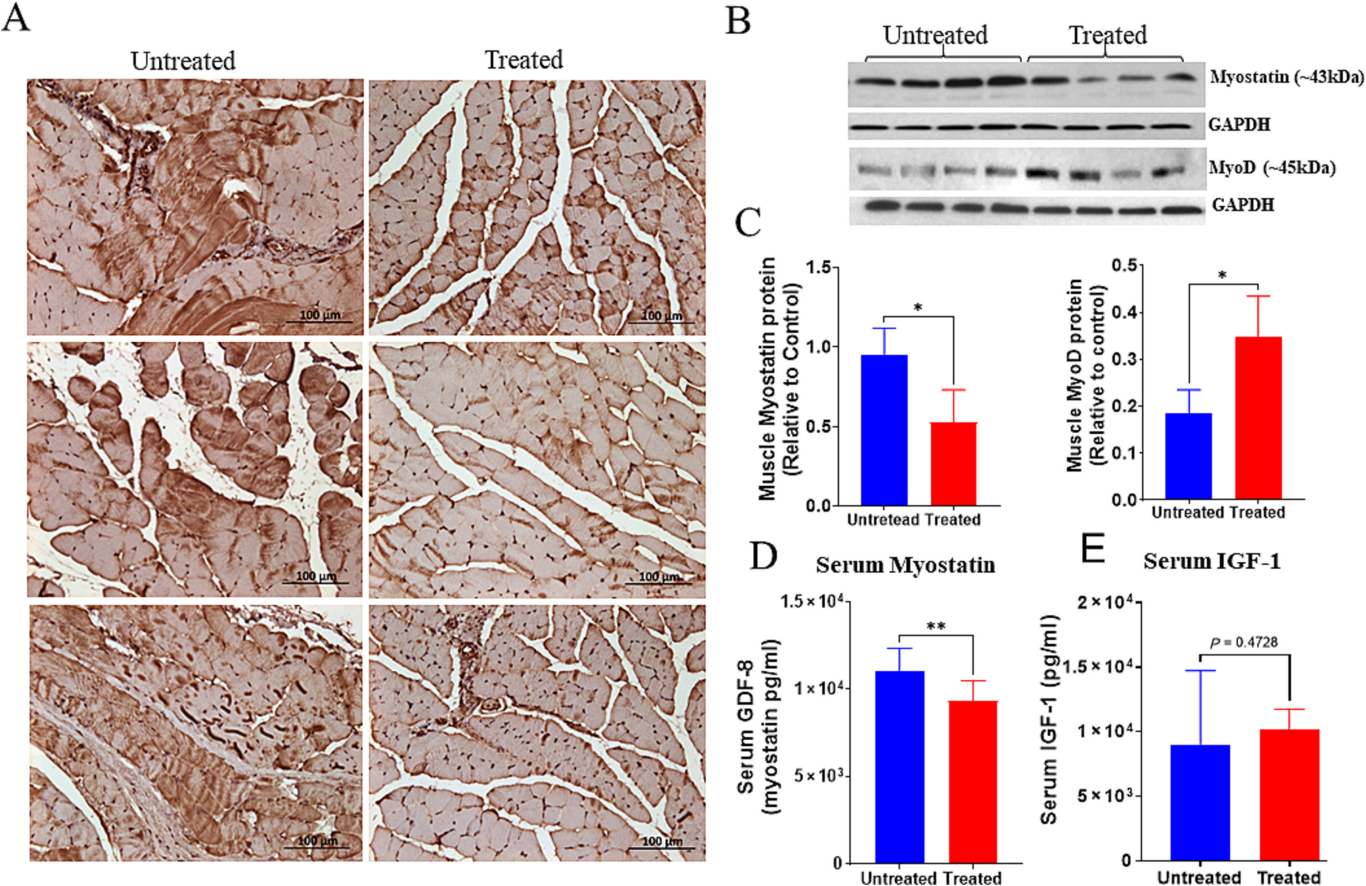
DFP treatment decreased the expression of myostatin in muscles and serum. (A) Immunohistochemistry of gastrocnemius muscle from untreated and DFP‐treated mice for myostatin expressions (*n* = 3). Representative images of each mouse from both groups are shown. (B) Western blot analysis for expressions of myostatin and MyoD, in gastrocnemius muscles from untreated and DFP‐treated mice (*n* = 4 mice/group). (C) Graphs show the quantitative results of myostatin and MyoD. Protein bands were normalized with GAPDH. Results are presented as expressions relative to control (*p < 0.05, *p = 0.017, respectively). (D) Histograms show the levels of myostatin and IGF‐1 (E) in untreated and DFP‐treated mice serum, **p = 0.003 (*n* = 12 for each group). Data are shown as mean ± SD. Asterisks denote levels of significant differences between groups as calculated with a two‐tailed, unpaired Student's *t* test.

To further evaluate the role of iron in sarcopenia, we examined the effect of deferiprone on TGF superfamily member myostatin, a factor that promotes catabolism and leads to skeletal muscle atrophy. As shown in Figure 4B, myostatin protein levels in gastrocnemius muscle were higher in untreated group of mice and decreased by ~1.8‐fold after treatment (*p* < 0.05, Figure 4C) as analysed by Western blots. Similar results were observed by IHC staining of gastrocnemius muscle cross‐sections (Figure 4A) as well as by serum myostatin (GDF‐8) ELISA measurements, showing circulating Mstn protein decreased from 11 021 ± 1316 to 9356 ± 1134 pg/mL with DFP treatment (Figure 4D, *p* = 0.0031). Decreased immunoreactivity for myostatin protein was observed in the muscle sections of wild type old mice (Figure S8B). Further, we checked the effect of DFP on the regenerative capacity of muscle cells. As shown by western blot analysis, aging‐associated pattern was observed for MyoD protein levels in untreated mice (Figure 4B), which showed lower levels in the gastrocnemius muscles compared with treated mice; it was increased by~1.9 fold after treatment (Figure 4B,C, *p* = 0.017). Growth hormone and IGF‐1 are anabolic hormones that induce muscle growth by increasing protein synthesis [30]. Hormones regulated by the endocrine system also play an important role in the balance between skeletal muscle anabolism and catabolism. We therefore examined the effect of DFP on IGF‐1 in the serum of untreated and treatment groups. As shown by ELISA analysis, in Figure 4E, the untreated group had lower levels of IGF‐1 (8952 ± 5779 pg/mL), which was increased, but the change was not statistically significant after DFP treatment **(**10 212 ± 1511 pg/mL, *p* = 0.472).

### 3.6 Effect of Iron Chelator on Cytokines

**FIGURE 5:**
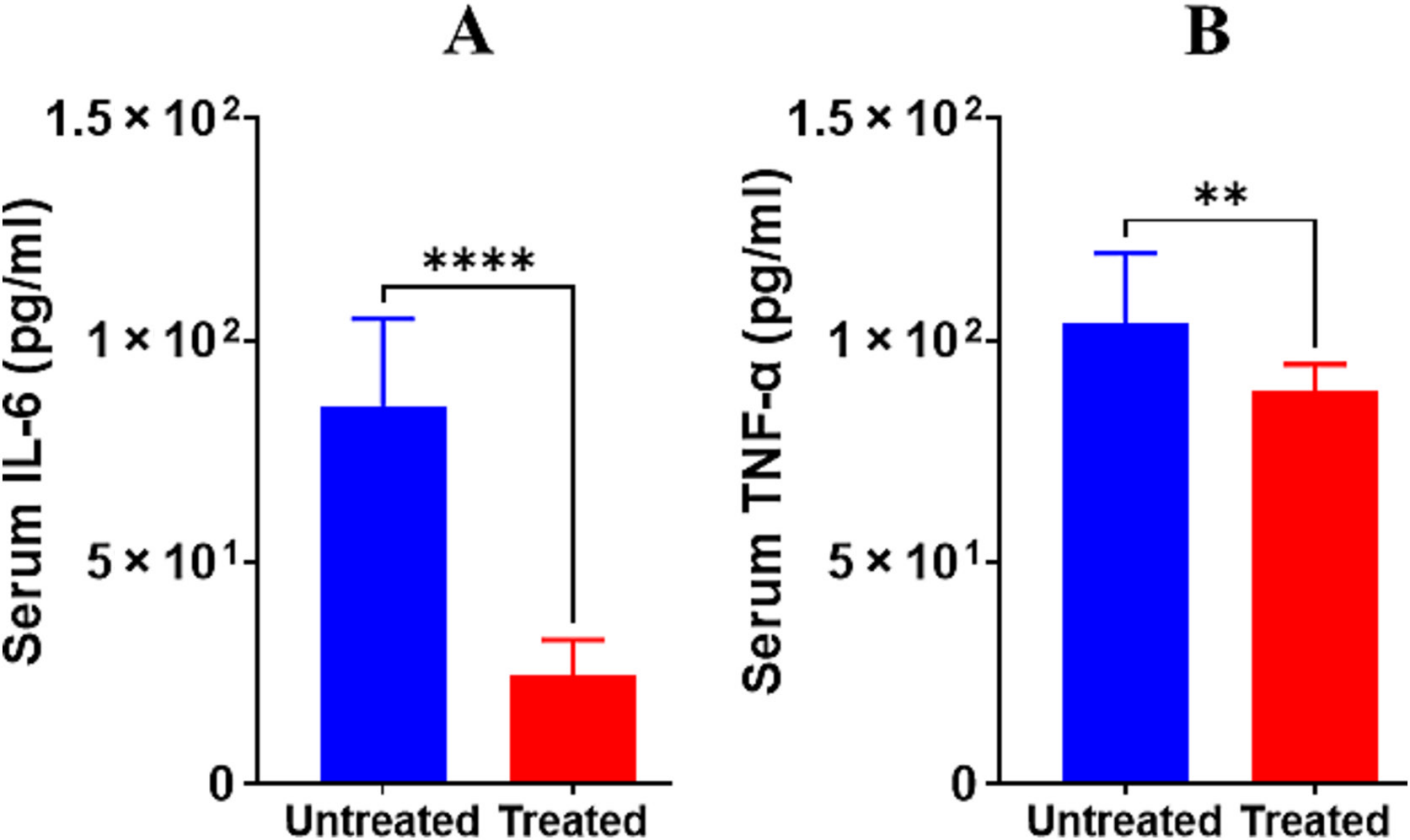
Reduction of inflammatory cytokines after DFP treatment. Histograms show the data of the plasma/serum TNF‐ά (A) IL‐6 (B) for the mice from klotho^−/−^ control and treated with 25 mg/kg deferiprone for 8 weeks (n = 12 each group). ***p < 0.001, **p < 0.01 compared to untreated mice as calculated with a two‐tailed, unpaired Student's *t* test.

Inflammatory activity is a characteristic part of the pathologic processes in several age‐associated disorders, including sarcopenia (S15) [31, 32]. Hence, we assayed the release of pro and anti‐inflammatory cytokines, TNF‐α and IL‐6. As shown by ELISA, we found that circulating levels of TNF‐α and IL6 in untreated group were increased compared with the results from treated mice (Figure 5A,B). DFP treatment decreased the cytokine levels from 103.73 ± 15.93 to 88.82 ± 5.80 pg/mL and 85.17 ± 19.65 to 24.52 ± 7.88 pg/mL, in TNF‐alpha and IL‐6, respectively (*p* = 0.005 for TNF‐α and *p* < 0.0001 for IL‐6, Figure 5A,B). Our results complement the finding of myostatin levels before and after DFP treatment.

### 3.7 Iron Chelation Decreases the Oxidative DNA Damage and Reduces Apoptosis in Skeletal Muscles of Klotho^−/−^ Mice

#### 3.7.1 8‐Hydroxy‐2 ‐Deoxy Guanosine (8‐OHdG) Immunohistochemistry

**FIGURE 6:**
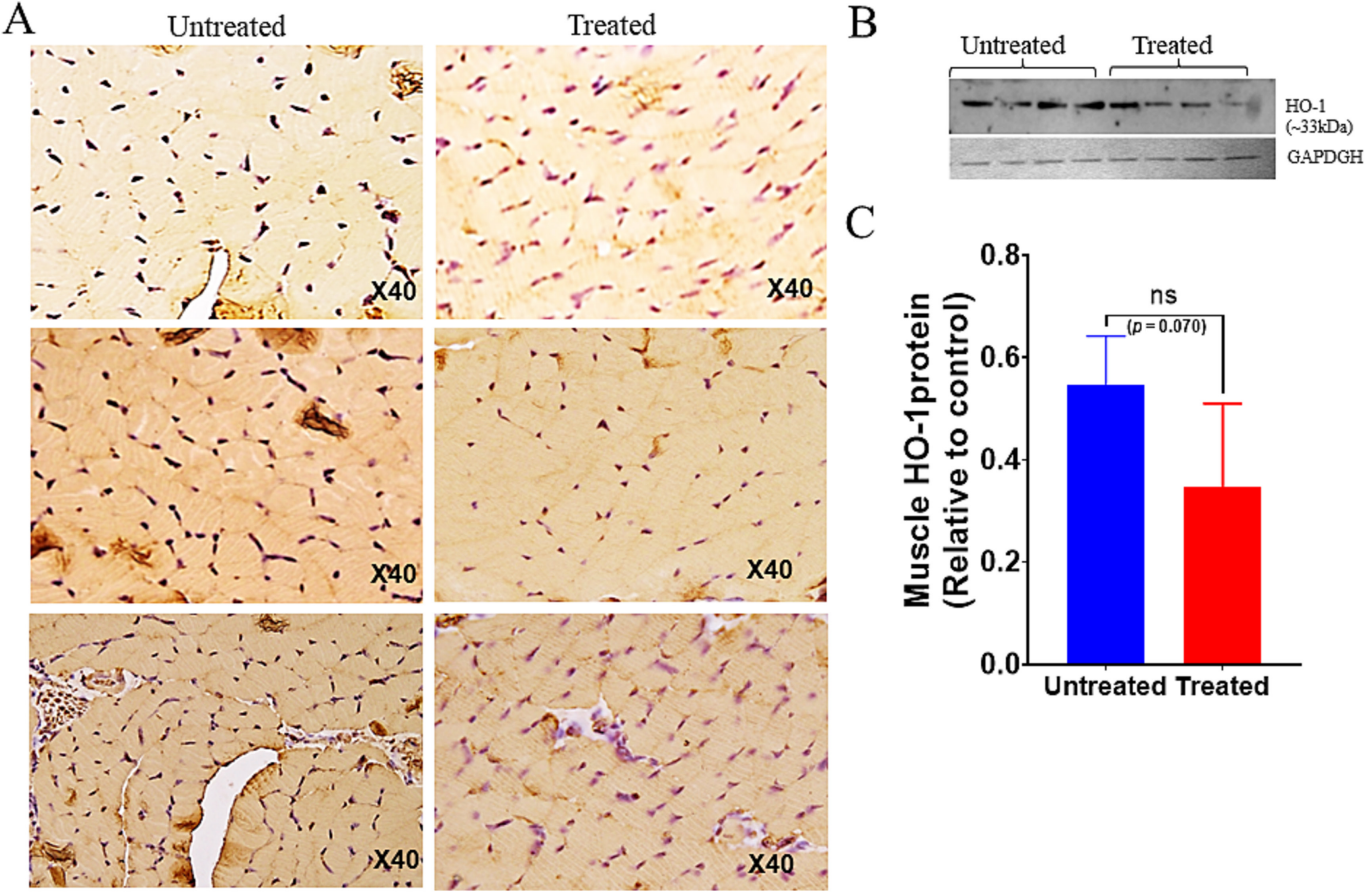
Deferiprone downregulates oxidative DNA damage in klotho^−/−^ mice. Panel (A) shows the immunostaining of 8‐hydroxy‐2‐deoxyguanosine in gastrocnemius muscles of untreated control and treated group. Positive signals were predominantly detected in the untreated muscle nuclei of cells. Representative images of *n* = 3 per group are shown (magnification X40). (B) Blot shows the western blot image of HO‐1(*n* = 4) from each group. (C) Quantitative analysis of western blot for HO‐1 (*p = 0.070). Protein bands were normalized with GAPDH. Results are presented as expressions relative to control. Data calculated with a two‐tailed, unpaired Student's *t* test.

**FIGURE 7:**
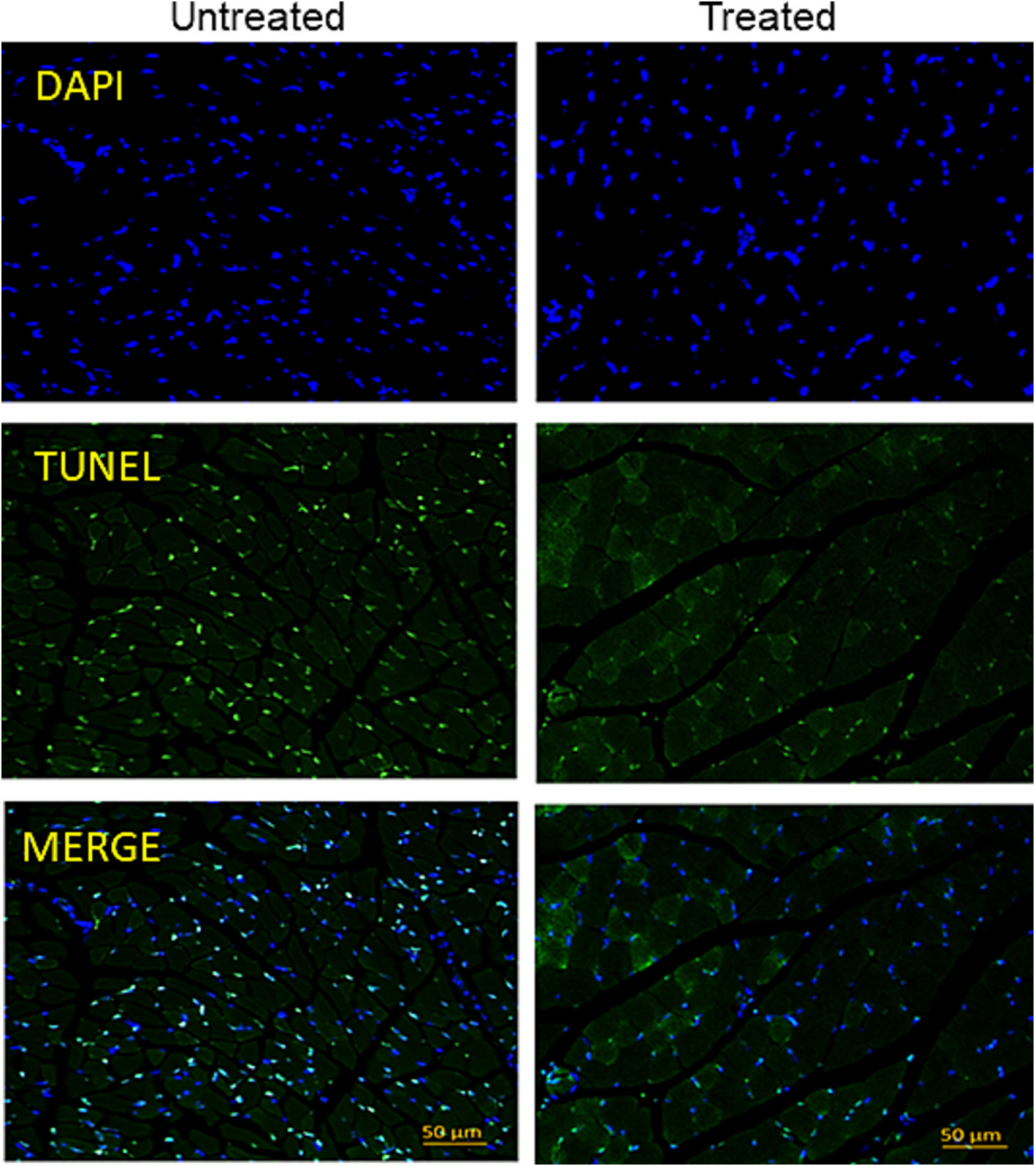
Iron chelation decreased cell apoptosis in muscle. Representative images of TUNEL assay show apoptotic cells in gastrocnemius muscles of untreated and treated mice. DAPI counterstaining nuclei. As observed, untreated mice showed a significantly higher number of tunnel‐positive nuclei compared to treated mice. Apoptotic cells are green (200X magnification). Scale bars are set at 50 μm for all.

We further investigated whether DFP treatment influences iron‐induced oxidative damage to DNA and apoptosis. We observed significantly increased immunoreactivity against 8‐OHdG in cross‐sections of gastrocnemius muscles of untreated mice compared with the treated groups (*p* = 0.0001, Figure 6A). Histochemical analysis revealed that muscles from untreated mice had strong 8‐oxo‐dG immunoreactivity in many cells and localized predominantly in the nucleus, compared to the treated group (Figure 6A). Heme oxygenase (HO‐1) is considered a critical cytoprotective mechanism that is activated during times of cellular stress such as inflammation, ischemia, hypoxia, hyperoxia, hyperthermia or radiation [33]. As shown by western blot analysis, the untreated group had induced HO‐1 protein levels, and expression was downregulated by ~1.6‐fold compared to untreated mice (*p* = 0.070, Figure 6C,D). Muscle sections were further analysed for apoptotic cell death by TUNEL staining. We found that in gastrocnemius muscle sections of DFP treated group had attenuated apoptosis compared to the untreated group (Figure 7). Muscles from treated mice had ~40%–50% less TUNEL‐positive nuclei per section (Figure 7). The overall number of TUNEL‐positive nuclei was low (~30% to 40%) and did correlate with the number of 8‐oxo‐dG immunoreactive cells (> 50%, Figure 6A,B) in treated muscles. Our findings showed that inhibition of myostatin by DFP treatment could attenuate apoptosis in skeletal muscles of klotho^−/−^ mice. These findings were supported by a 1.8‐fold lower myostatin expression in DFP‐treated mice (*p* < 0.05, Figure 5C).

### 3.8 Effect of DFP on CBC and Other Biochemical Parameters

Table 1 shows the biochemical parameters among untreated and DFP‐treated groups at the end of the therapy. Dose of DFP used did not result in anaemia, as there was no difference in the serum haemoglobin levels (Hb, g/dL), 10.54 ± 1.1 in untreated and 10.53 ± 1.2 treated groups at the end of the therapy. Mean serum phosphorus (PHOS) and calcium (CA, mg/dL) concentrations were higher in untreated groups. PHOS was significantly higher (13.94 ± 1.8 mg/dL) than DFP treated mice (10.9 ± 3.6, mg/dL). Hyperphosphatemia was significantly decreased by treatment in the DFP group (*p* < 0.05), with minimal effects on serum calcium, 8.5 ± 0.6 to 8.0 ± 4.0 mg/dL in untreated and treated groups, respectively. Hyperphosphatemia has been linked to aKlotho (aKL) deficiency, which is correlated with increased serum fibroblast growth factor‐23 (FGF23). As shown in Table 1, serum intact FGF23 was elevated in untreated mice (233.07 ± 48.77 pg/mL) and was reduced to 69.39 ± 33.95 pg/mL (Table 1) (*p* < 0.0001) in the treated mice. Klotho, the coreceptor of the fibroblast growth factor 23 (FGF‐23), can interfere with calcium and phosphate metabolism. The serum level of ALP was significantly higher in the untreated group (184.6 ± 62.9 vs. 75.14 ± 25.5, U/L, *p* < 0.001), compared to treated respectively (Table 1). Low Na^+^ and K^+^ along with slightly high creatinine (0.2 ± 0.1 vs. 0.17 ± 0.04 mg/dL), and significantly higher blood urea nitrogen (53.2 ± 23.3 vs. 25.3 ± 17.1, mg/dL *p* < 0.001), indicate that untreated mice were dehydrated and had some muscle loss as shown by their water consumption and muscle weights (Figure 2B,C and Table 1). There was no significant difference in other parameters tested between the two groups of mice.

**TABLE 1:** Blood parameters of liver and kidney function test in the serum of klotho^−/−^ mice.

	Untreated	Treated
Hb (g/dL)	10.54 ± 1.1	10.53 ± 1.2
RBC (M/μL)	8.71 ± 1.5	8.83 ± 0.85
HCT (%)	43.34 ± 5.8	43.65 ± 6.2
CA (mg/dL)	8.5 ± 0.6	8.0 ± 4.0
PHOS (mg/dL)	13.94 ± 1.8	10.9 ± 3.6*
FGF23 (pg/mL)	475.60 ± 234.41	192.44 ± 125.15*
ALP (U/L)	184.6 ± 62.9	75.14 ± 25.5***
GLU (mg/dL)	121 ± 29.7	134 ± 43.0
NA+(mmol/L)	162 ± 11.0	157 ± 1.0
K + (mmol/L)	4.6 ± 1.2	5.12 ± 0.4*
TP (g/dl)	4.4 ± 1.7	4.9 ± 0.4
TBIL (mg/dL)	0.32 ± 0.04	0.32 ± 0.04
BUN (mg/dL)	53.2 ± 23.3	25.3 ± 17.1***
CRE (mg/dL)	0.2 ± 0.1	0.17 ± 0.04

*Note:* Mice were treated (*n* = 7) with 25 mg/kg BW of deferiprone for 8 weeks as above. Serum analyses were done with VetScan Vs2 (comprehensive diagnostics) in CAVHS. The data are presented as means ± SDM.

Abbreviations: ALP, alkaline phosphatase, BUN, blood urea nitrogen, CA, calcium; FBF23, fibroblast growth factor23; GLU, glucose; Hb, haemoglobin; PHOS, phosphorus; TBIL, total bilirubin; TP, total protein.

* *p* < 0.05.

** *p* < 0.01.

*** *p* < 0.001 versus untreated (*n* = 5) group by unpaired *t* test.

## 4 Discussion

Our study presents the novel observation that an iron chelator inhibits age‐associated changes of skeletal muscle sarcopenia in klotho^−/−^ mice and that treatment with deferiprone resulted in significant prolongation of the life span with median survival of mice increased to 108 days compared to 63 days in untreated controls mice (Figure 2D). In our study, deferiprone given at a daily dose of 0.25 mg/kg/day reduced total and catalytic iron and tissue iron. Importantly, in doses used, it did not result in anaemia with levels of Hct and Hb being similar in treated and untreated groups.

Mice given deferiprone maintained body weight and muscle mass (based on weight). In humans, the structural changes responsible for age‐related muscle atrophy and decline in muscle strength are related to the decline in the cross‐sectional fibre area (up to 25%–30%) [2, 34] (S16) and fibre denervation and fibre number loss [35]. Histological examination of gastrocnemius muscles revealed more muscular dystrophy at 12 weeks of age—characterized by myonecrosis, central nucleation and small atrophic fibres (Figures S4 and S5)—in untreated mice than in the treatment group of mice. DFP treatment increased the myosin heavy chain protein content of skeletal muscle as evident in the expression of myosin in western blot results. The myogenic differentiation marker, MyHC, is the major structural protein in myotubes (S17) [36]. The DFP‐treated mice muscles exhibited an increased expression of MyHC, compared with the untreated mice.

Myostatin, a TGF superfamily member, is a factor that promotes catabolism, leading to skeletal muscle atrophy. Myostatin has been implicated in several wasting conditions including sarcopenia and cachexia [37]. Inhibition of myostatin has been shown to improve muscle mass in aging (S15–S21) [38, 39]. In our study, treatment of mice with DFP showed a decrease in muscle and serum myostatin protein. It is well known that MyoD is one of the proteins that is required for muscle proliferation and differentiation [40]. We found decreased expressions of muscle MyoD in untreated mice muscle, and it was increased significantly after DFP treatment. These findings of increased expression of the myogenic protein, and inhibition of myostatin expression of deferiprone further supports the observed effect of deferiprone on muscle mass.

In several systems, the amount of free‐radical generation is related to the amount of catalytic iron present [40]; its role has been demonstrated in many disease states including acute and chronic kidney disease [41] (S3), cardiovascular disease [42] (S22, S23) and neurodegenerative disorders (S24, S25) [43]. Thus, its role in disease processes appears to be a common theme of cellular injury. The potential mechanisms by which iron induces sarcopenia include its ability to induce oxidative stress and inflammation, as well as its ability to directly induce muscle injury and fibrosis.

Oxidative damage has been proposed as one of the major contributors to the skeletal muscle decline occurring with aging (S8, S26). Treatment with deferiprone significantly reduced immunoreactivity against 8‐OHdG in cross sections of muscles compared to untreated mice. The untreated group had induced protein levels, and expression of HO‐1 protein was significantly downregulated by ~1.8‐fold compared to untreated. Muscles from treated mice had less apoptotic cell death as measured by TUNEL‐positive nuclei per section. Our findings showed that inhibition of myostatin by DFP treatment effectively decreased apoptosis in skeletal muscles of klotho^−/−^ mice. These findings were supported by lower myostatin expression in DFP‐treated mice.

Inflammatory activity is a characteristic part of the pathologic processes in several age‐associated disorders, including sarcopenia (S12) [30, 31, 32]. Inflammatory cytokines can cause a low‐level chronic inflammation, which contributes to the activation of protein degradation in muscle (S27) [44]. Our results demonstrate that circulating levels of TNF‐α and IL6 in the treated group were lower compared with treated mice. Our results complement the finding of myostatin levels before and after DFP treatment.

The process of sarcopenia has devastating ramifications for senior citizens as they experience the loss of muscle mass and strength to the point at which they can no longer be independent. Our studies support the important role of iron in the pathophysiology of skeletal muscle sarcopenia. Although klotho^−/−^ mice are useful for examining the pathophysiology of iron‐induced muscle sarcopenia related to aging, premature aging animal models might not completely recapitulate the natural course of aging in humans. Preliminary studies with 28‐month‐old wild type mice data supported our finding with Klotho^−/−^ mice. However, additional studies are needed to evaluate the efficacy in the human environment. Nonetheless, to our knowledge, this is the first study to demonstrate an association between iron, specifically catalytic iron, and age‐related skeletal muscle sarcopenia and provide evidence that an iron chelator can improve age‐related muscle sarcopenia in a mouse model. The outcome of our study has the potential for the future development of a novel therapy for age‐associated sarcopenia and related muscle weakness and degradation. This will be an important endeavour in improving the quality of life for the elderly.

## 5 Conclusions

The cause of sarcopenia or decline in muscle mass and function with aging remains unclear and contributes to functional limitations in the elderly. The treatment of sarcopenia has been challenging and is mostly concentrated on prevention by exercise, with nutritional support and use of anabolic medication. There remains a significant need for a novel therapy that can improve sarcopenia and related problems in aging. Our study presents the novel observation that indicates role of iron in the pathophysiology of sarcopenia in kl/kl mice and identifies excess labile (catalytic, CatFe) iron as one of the key factors of developing sarcopenia with aging. Thus, iron chelation has the potential for the development of a novel therapy for sarcopenia.

## Conflicts of Interest

The authors declare no conflicts of interest.

## Supporting information


**Figure S1.** Study design and treatment schedule for deferiprone. Untreated mice did not survive beyond 14 weeks. Treated mice survived more than 18 weeks.
**Figure S2.** Phenotype difference in 12 weeks old klotho mutant (a) and wild type (b)
**Figure S3.** Genotyping details for *klotho,* wild‐type, klotho+/− and knockout genes. Panels show the primer sequences, PCR thermal cycling protocol, and representative agarose gel images for each genotype. Extract‐N‐Amp Tissue PCR kit from Sigma was used for DNA extraction and PCR. 10 μL of DNA (per sample) was used to run the on 1.5% agarose gel for final product 455 bp is klotho−/−.
**Figure S4.** H&E staining gastrocnemius sections. Increased pathological changes are observed in untreated klotho−/−section, including centrally located nuclei, Arrows show, centrally located nuclei and infiltration of inflammatory cells. Significantly a smaller number of centrally located nuclei were observed in the muscle's sections of treated mice. All images are at 200X magnification.
**Figure S5.** Klotho−/−mice untreated and treated with deferiprone for 8 weeks. Skeletal muscles from gastrocnemius were stained for laminin by IHC to check the integrity of muscle fibres. As shown in figure, there was increased in the laminin immunoreactivity, after treatment muscle fibre structures looks more uniform whereas decrease in laminin in untreated muscles and in some fibres complete loss of laminin immunoreactivity was observed (arrow heads). All images are at 400X magnification.
Figure S6. Full gel images of western blots.
**Figure S7. Iron chelation with DFP reduced iron accumulation muscles of old mice**. 26 months old C57BL/6 male mice were treated with 100 mg/kg body wt. of DFP for 10 months. Prussian Blue staining was done for iron. Bright field images of gastrocnemius muscles from untreated and treated mice. Arrows show the positive staining for presence of iron (blue). More iron accumulation was seen in untreated mice than treatment group. Sections were counterstained with Nuclear Fast Red. (*n* = 2, representative images of each mouse from both groups are shown). All images are at X40.
**Table S1.** Primary antibodies were used in this study. IHC‐immunohistochemistry, WB‐western blot.

## Data Availability

All study data are included within the paper and the Supporting Information.
