# Supplementary material for: Iron Chelation Prevents Age‐Related Skeletal Muscle Sarcopenia in Klotho Gene Mutant Mice, a Genetic Model of Aging
Source: J Cachexia Sarcopenia Muscle. 2025 Jan 10;16(1):e13678. doi: 10.1002/jcsm.13678 (PMC11724147; doi:10.1002/jcsm.13678)
Supplement: Supplementary file 1 — Figure S1. Study design and treatment schedule for deferiprone. Untreated mice did not survive beyond 14 weeks. Treated mice survived more than 18 weeks. Figure S2. Phenotype difference in 12 weeks old klotho mutant (a) and wild type (b) Figure S3. Genotyping details for klotho, wild‐type, klotho+/− and knockout genes. Panels show the primer sequences, PCR thermal cycling protocol, and representative agarose gel images for each genotype. Extract‐N‐Amp Tissue PCR kit from Sigma was used for DNA extraction and PCR. 10 μL of DNA (per sample) was used to run the on 1.5% agarose gel for final product 455 bp is klotho−/−. Figure S4. H&E staining gastrocnemius sections. Increased pathological changes are observed in untreated klotho−/−section, including centrally located nuclei, Arrows show, centrally located nuclei and infiltration of inflammatory cells. Significantly a smaller number of centrally located nuclei were observed in the muscle's sections of treated mice. All images are at 200X magnification. Figure S5. Klotho−/−mice untreated and treated with deferiprone for 8 weeks. Skeletal muscles from gastrocnemius were stained for laminin by IHC to check the integrity of muscle fibres. As shown in figure, there was increased in the laminin immunoreactivity, after treatment muscle fibre structures looks more uniform whereas decrease in laminin in untreated muscles and in some fibres complete loss of laminin immunoreactivity was observed (arrow heads). All images are at 400X magnification. Figure S6. Full gel images of western blots. Figure S7. Iron chelation with DFP reduced iron accumulation muscles of old mice. 26 months old C57BL/6 male mice were treated with 100 mg/kg body wt. of DFP for 10 months. Prussian Blue staining was done for iron. Bright field images of gastrocnemius muscles from untreated and treated mice. Arrows show the positive staining for presence of iron (blue). More iron accumulation was seen in untreated mice than treatment group. Sections were count [file JCSM-16-e13678-s001.docx]

**Supporting Information for**

**Iron chelation prevents age-related skeletal muscle sarcopenia in klotho gene mutant mice, a genetic model of aging**

Chhanda Bose* ^a, b, c^_,_ Judit Megyesi ^b^, Oleg Karaduta ^a, b, e^, Sharda P Singh ^a, b, d^, Sundararaman Swaminathan ^a, b^, Sudhir V Shah* ^a, b^

^a^ Central Arkansas Veterans Healthcare System, Little Rock, Arkansas, 72205, USA; ^b^ University of Arkansas for Medical Sciences, Little Rock, Arkansas, 72205 USA. ^c^ Pharmacology and Neuroscience department, Texas Tech Health Sciences Center, Lubbock, Texas, 79430, USA**.** ^d^ Internal Medicine Department, Texas Tech Health Sciences Center, Lubbock, Texas, 79430, USA**.**^e^ Department of Biochemistry and Molecular Biology, UAMS, Little Rock, AR 72205, USA.

*Corresponding authors

**Email:**  [Chhanda.bose@ttuhsc.edu](mailto:Chhanda.bose@ttuhsc.edu);

[ShahSudhirV@uams.edu](mailto:ShahSudhirV@uams.edu)

**This PDF file includes:**

Supplemental Figures S1 to **S8**

Supplemental Tables S1

Supporting text (Supplemental Materials and Methods)

Supplemental References

*Running title: Prevention of age-related skeletal muscle sarcopenia*

**Supplemental figures and legends**

**
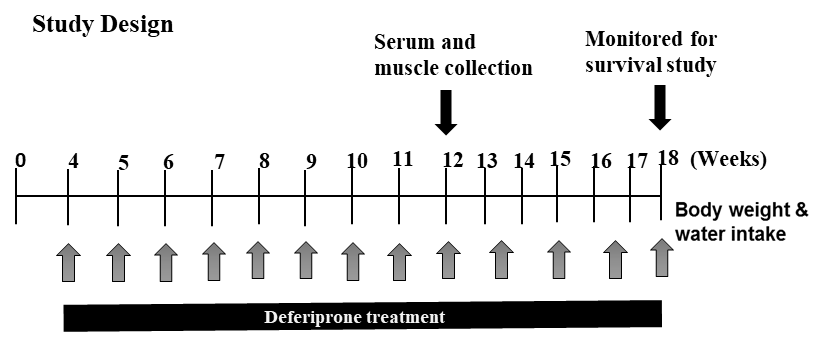
**

**Figure S1.** Study design and treatment schedule for deferiprone. Untreated mice did not survive beyond 14 weeks. Treated mice survived more than 18 weeks.


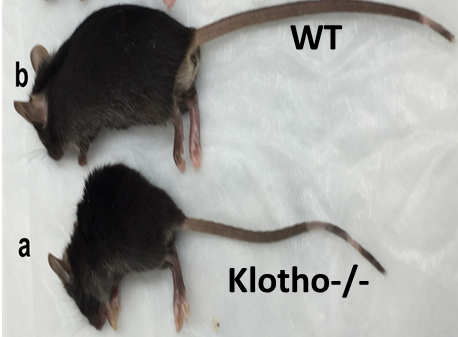


**Figure S2.** Phenotype difference in 12 weeks old klotho mutant (a) and wild type (b)


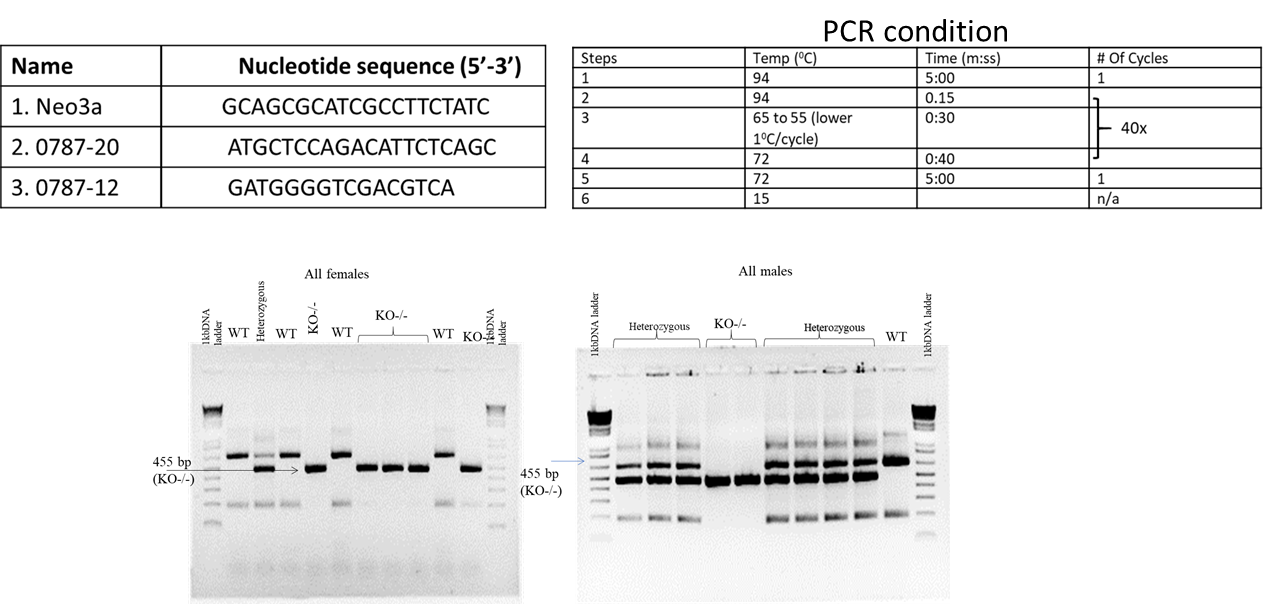


**Figure S3.** Genotyping details for *klotho,* wild-type, klotho+/- and knockout genes. Panels show the primer sequences, PCR thermal cycling protocol, and representative agarose gel images for each genotype. Extract-N-Amp Tissue PCR kit from Sigma was used for DNA extraction and PCR. 10 µl of DNA (per sample) was used to run the on 1.5% agarose gel for final product 455bp is klotho-/-.


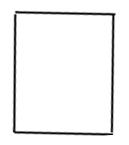

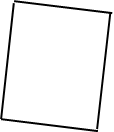

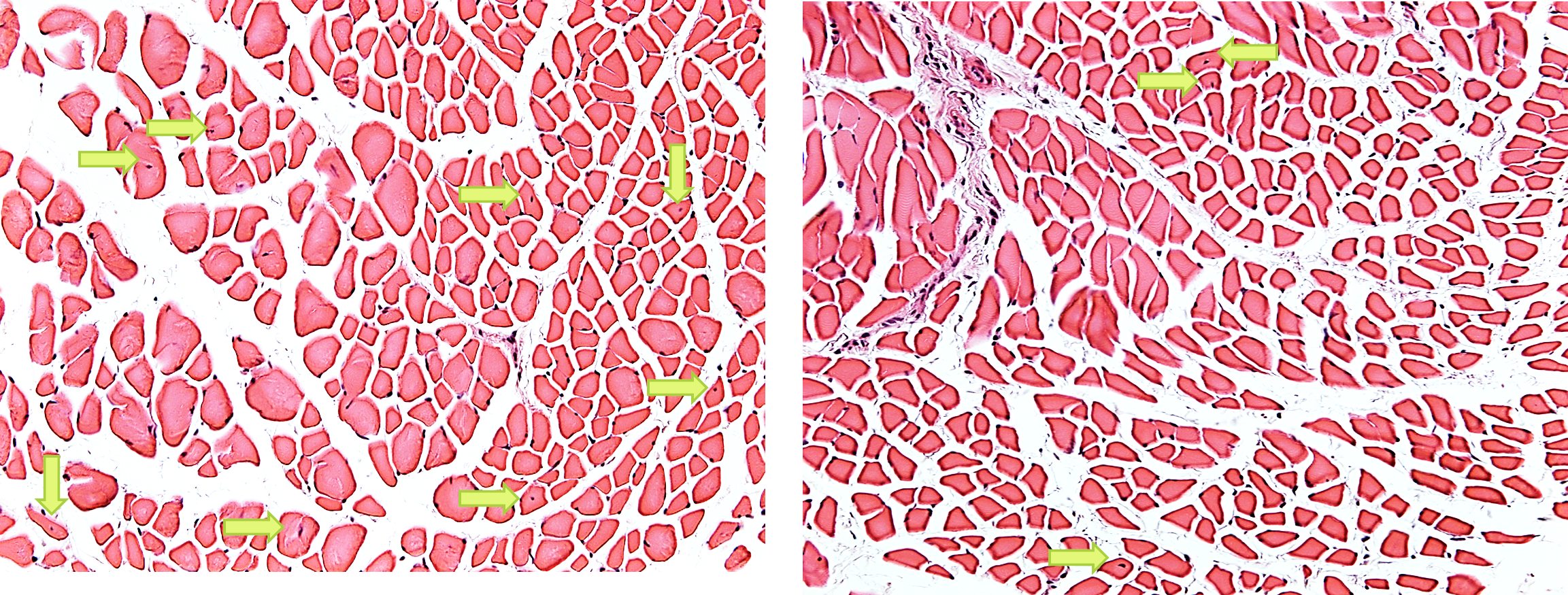

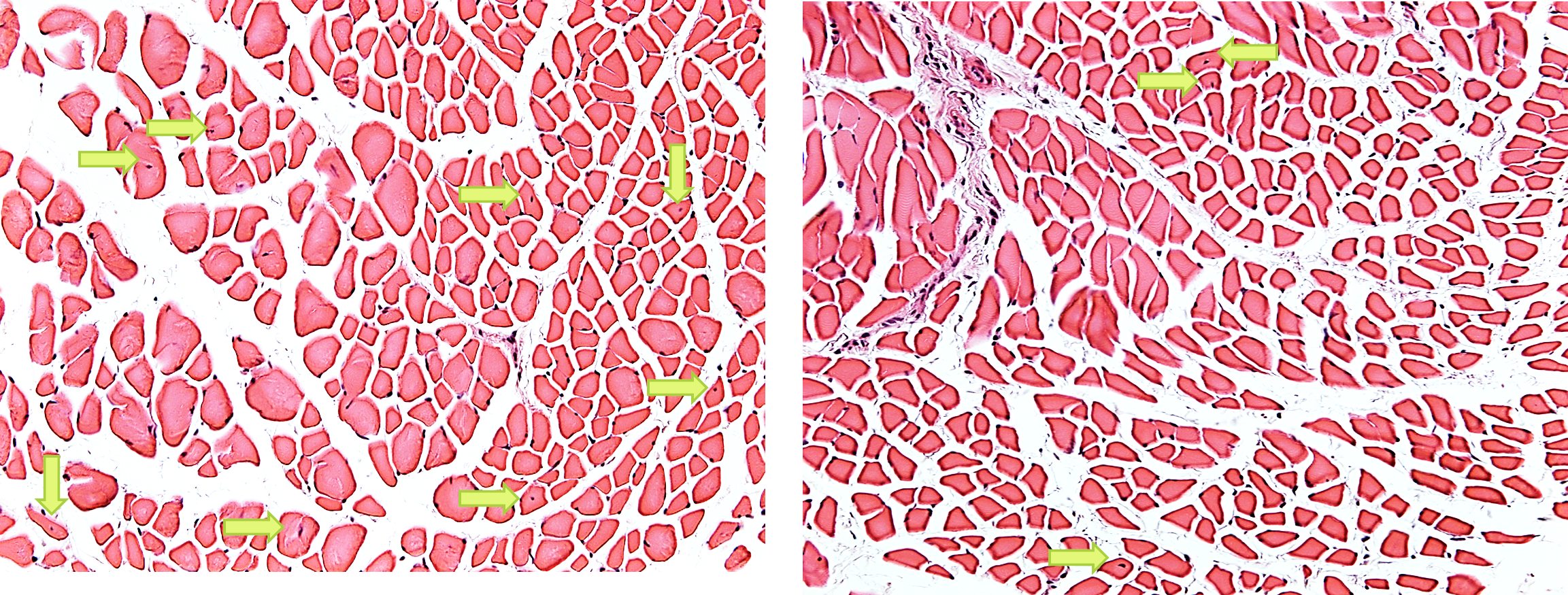

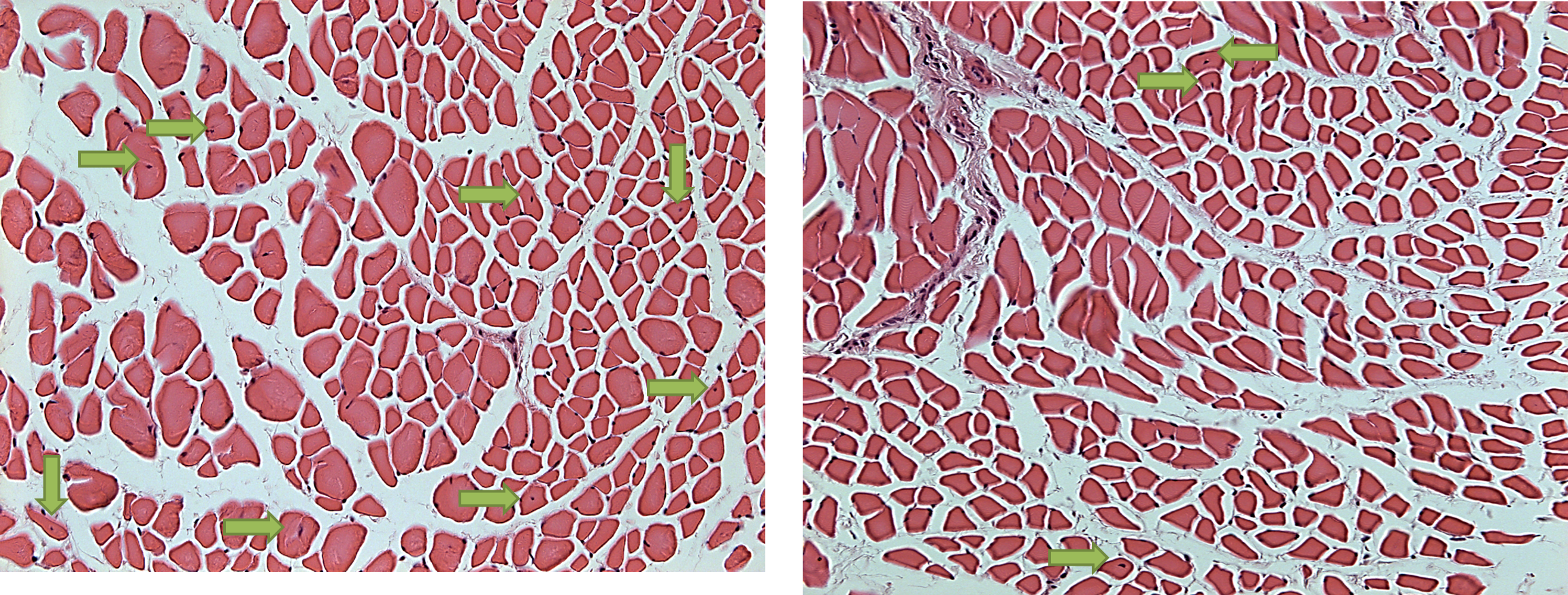

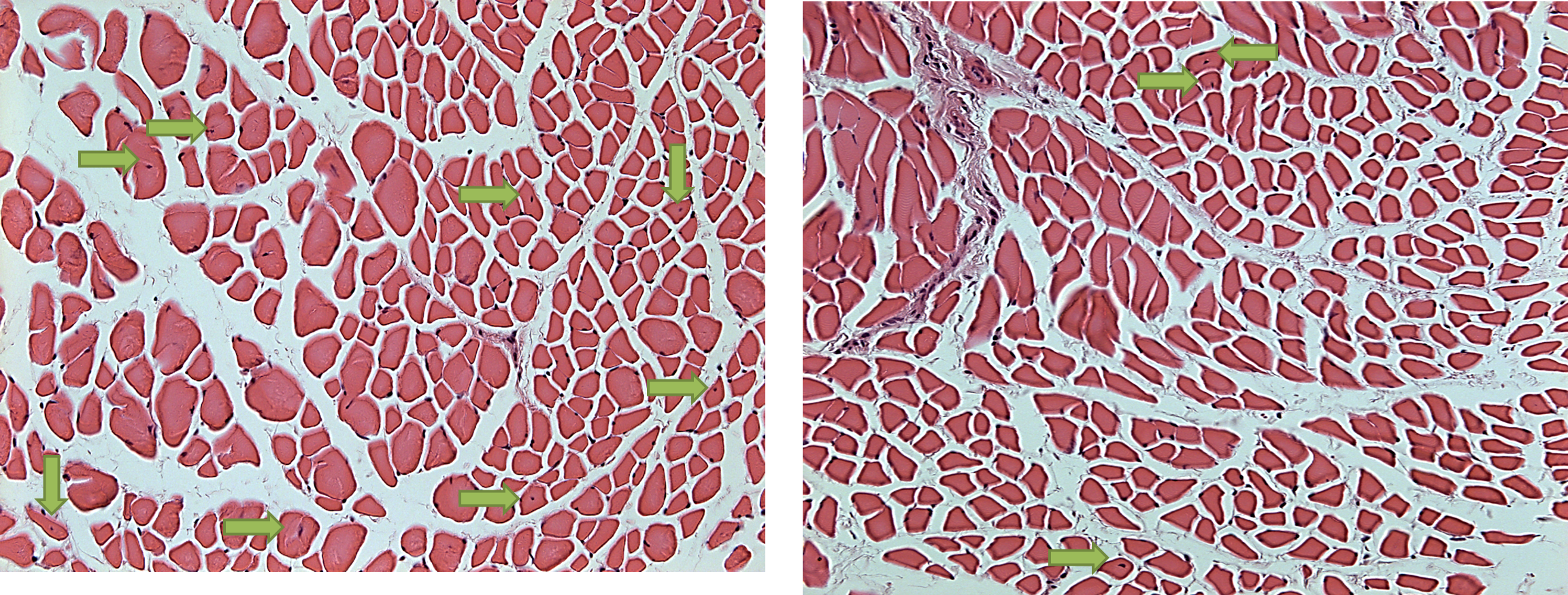


Untreated

X20

Treated

X20

**Figure S4.** H&E staining gastrocnemius sections. Increased pathological changes are observed in untreated klotho-/-section, including centrally located nuclei, Arrows show, centrally located nuclei and infiltration of inflammatory cells. Significantly a smaller number of centrally located nuclei were observed in the muscle's sections of treated mice. All images are at 200X magnification.


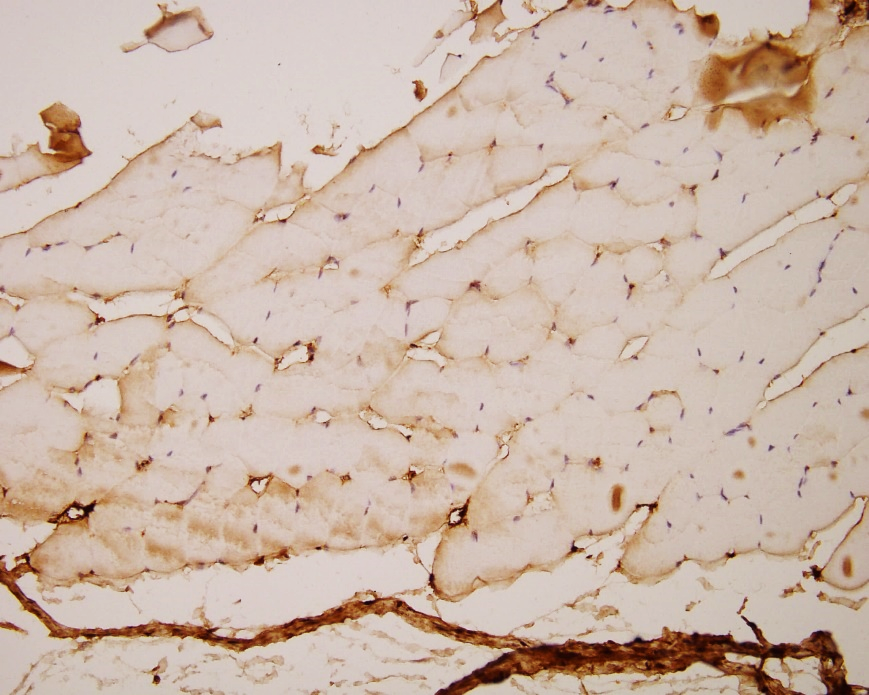


x40


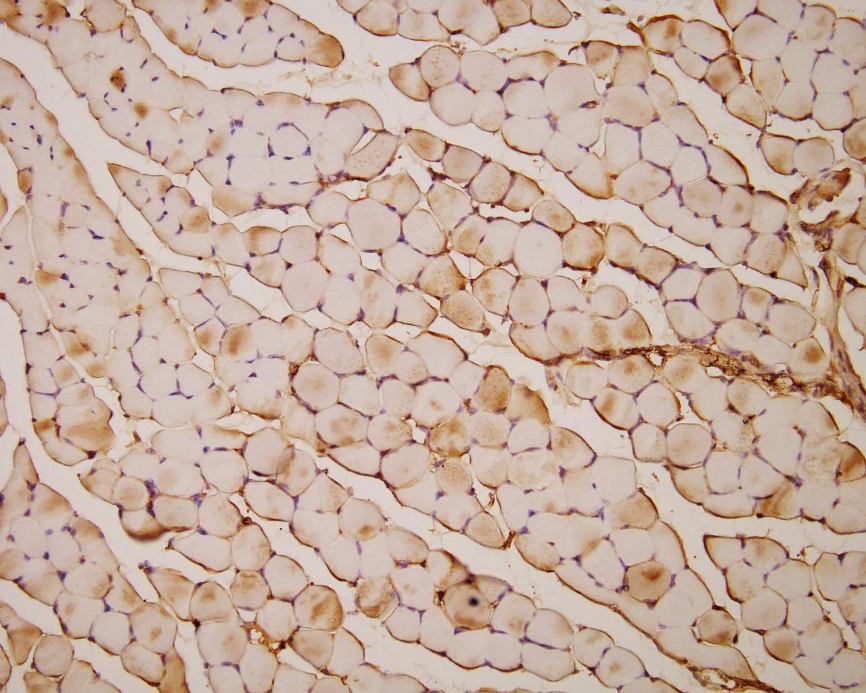


Treated

x40

Untreated

**Laminin**

**Figure S5.** Klotho-/-mice untreated and treated with deferiprone for 8 weeks. Skeletal muscles from gastrocnemius were stained for laminin by IHC to check the integrity of muscle fibers. As shown in figure, there was increased in the laminin immunoreactivity, after treatment muscle fiber structures looks more uniform whereas decrease in laminin in untreated muscles and in some fibers complete loss of laminin immunoreactivity was observed (arrow heads). All images are at 400X magnification.

**Figure S6. Full gel images of western blots.**


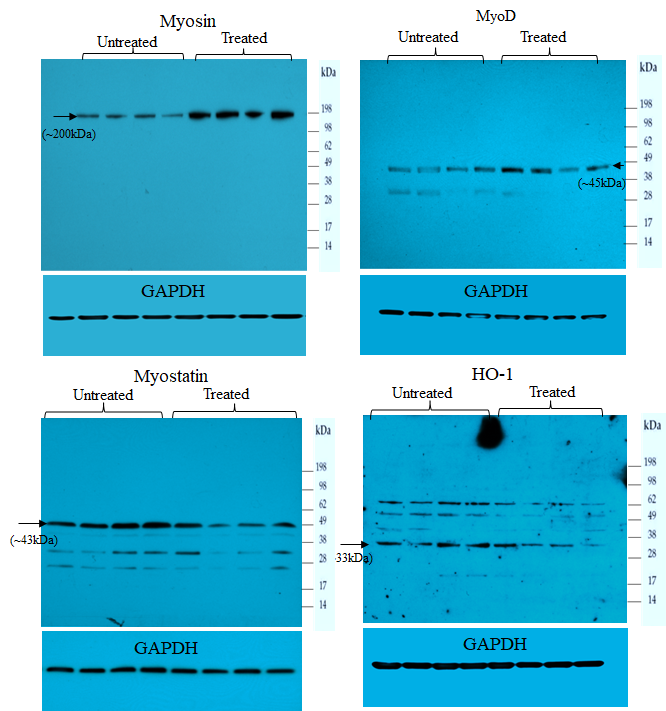


**Perl’s Prussian blue staining for iron accumulation in muscles**

**of old wild type C57BL/6 mice**


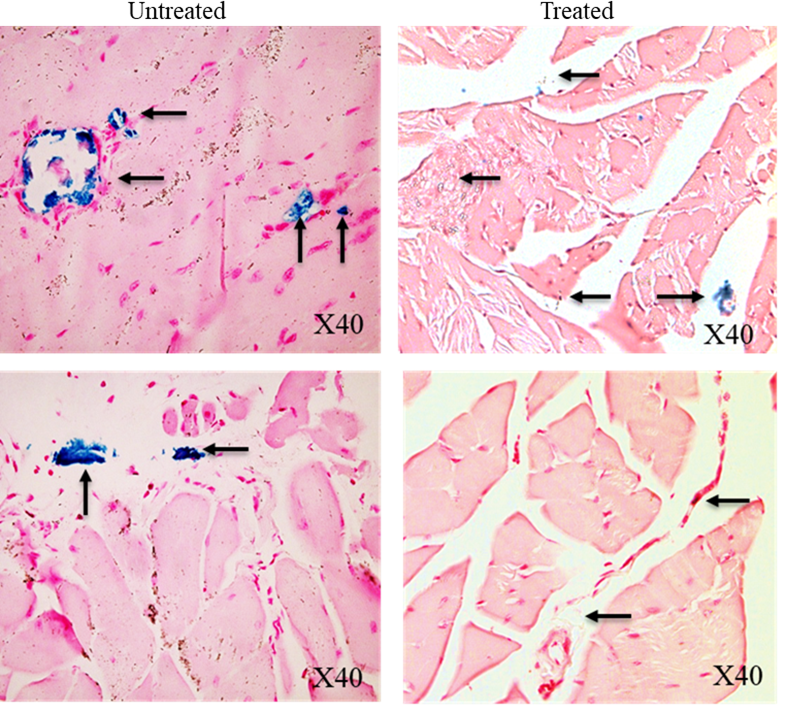


**Figure S7. Iron chelation with DFP reduced iron accumulation muscles of old mice**. 26 months old C57BL/6 male mice were treated with 100mg/kg body wt. of DFP for 10 months. Prussian Blue staining was done for iron. Bright field images of gastrocnemius muscles from untreated and treated mice. Arrows show the positive staining for presence of iron (blue). More iron accumulation was seen in untreated mice than treatment group. Sections were counterstained with Nuclear Fast Red. (n=2, representative images of each mouse from both groups are shown). All images are at X40.

**Muscle Myosin in wild type old mice**


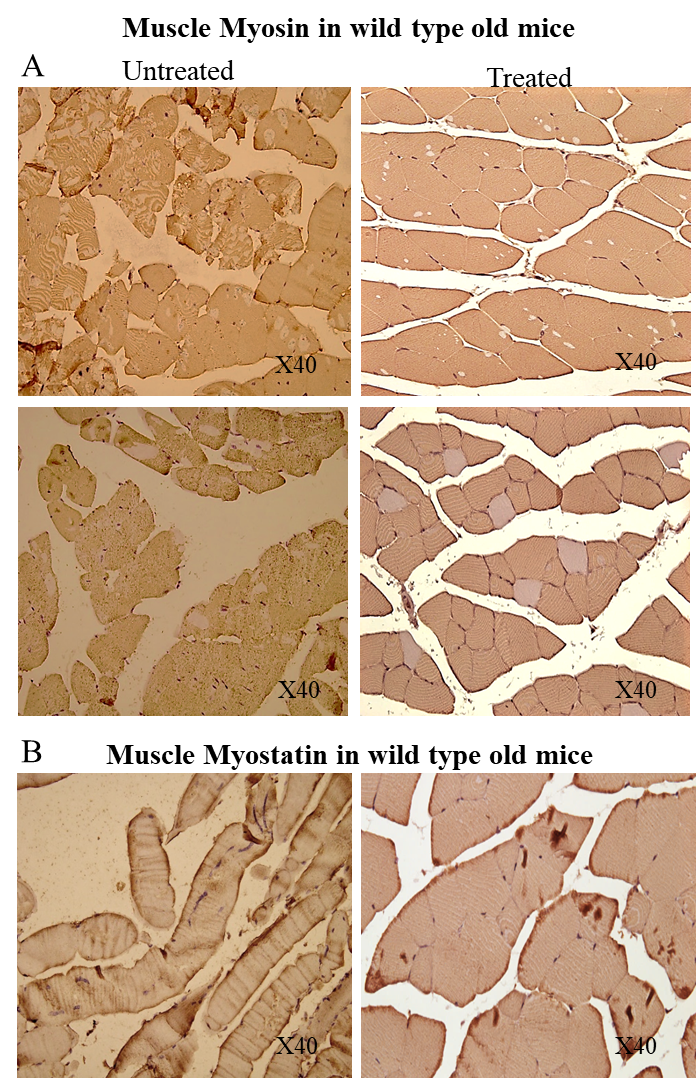


**Figure S8. Upregulation of contractile protein myosin heavy with iron chelation**. 26 months old male C57BL/6 mice were given 100mg/kg body wt. of DFP in drinking water for 10 weeks. Age of mice at the time of tissue collection was 28 months. (A) Representative IHC images of gastrocnemius muscles for myosin heavy chain in 28 months old wild type C57BL/6 mice (n=2 per group, representative images of each mouse from both groups are shown) (B) Downregulation of myostatin protein after DFP treatment. Representative IHC images are shown. All images at X40.


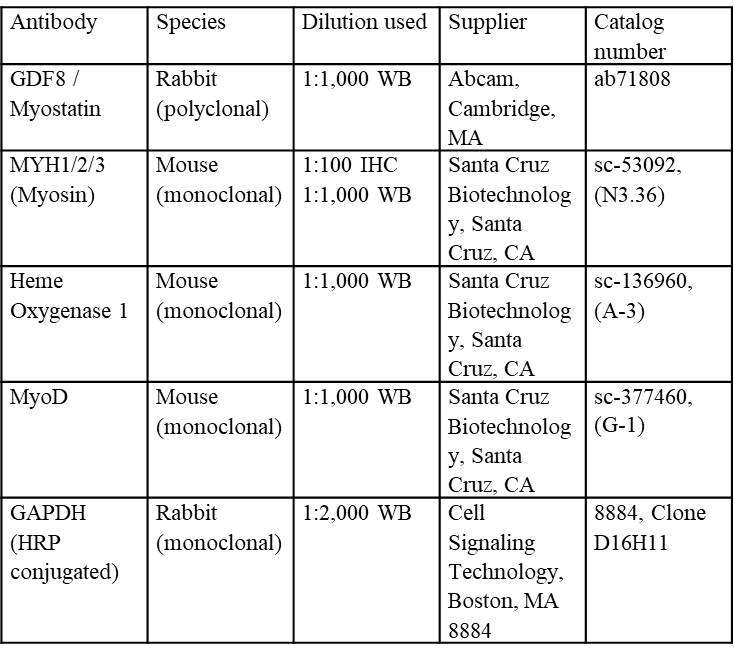


**Table S1.** Primary antibodies were used in this study. IHC-immunohistochemistry, WB-western blot.

**Supplementary Methods**

**Mouse husbandry**

*Klotho^+/-^* mice were housed on a 12-h light/dark cycle with ad libitum access to diets and water. Both groups of mice were given gel food because it is difficult for Klotho mice to gnaw and chew after they have started to display the aging phenotypes, and gel food helps them to eat the appropriate amount of food. Average dose intake was monitored by measuring the volume of water consumed in the group cage every alternate day. Body weights were recorded weekly. We performed serial experiments for each endpoint and the data were pooled to achieve statistical power analysis. Phenotypically *Klotho^-/-^* mice are significantly smaller compared to their wild-type littermates (*Figure* S2). Body weight of *Klotho^-/-^* mice ranges from 6-10 grams from 3-10 weeks of age, with reduced muscle and total body mass compared to wild-type mice, so gastrocnemius muscles were selected for analysis because they contain a variety of muscle fiber types, and the results may therefore be generalized to other muscles. In addition, the substantial amount of tissue in the gastrocnemius muscle allowed us to perform all the analyses on the same muscle. **Old wild type mice were used for preliminary studies with DFP treatment. Twenty-six months old C57BL/6 male mice were given 100mg/kg/body wt. of DFP in drinking water for 10 weeks. In the end of treatment, skeletal muscles were collected. Effect of iron chelation was examined for iron accumulation by Perl’s Prussian blue staining. Expression of muscle myosin heavy chain and myostatin were checked by IHC to examine the effect of iron chelation in age related sarcopenia. Detail methods are described in the method section.**

**Genotyping**

All pups were weaned and genotyped regularly at age 21–23 days. DNA was isolated from an approximately 2 mm piece of tail tip and DNA was isolated with Extract-N-Amp PCR kit (cat#XNAT2) following the protocol. In brief: 100µl of extraction solution and 25µl of tissue preparation solution were mixed for each sample and added to a clean micro centrifuge tube with tissue. Samples were incubated at room temperature for 10 minutes, followed by 3 min incubation at 95^0^C. 100µl of neutralization buffer was added to each tube. Samples were used immediately for PCR or stored in 4^0^C until ready to use. Genotyping was performed as suggested by mmrc UC Davis (Mutant mouse regional resource Center, UC Davis, CA), using recommended primer pairs specific for the klotho gene. Details on primer sequences and PCR cycling and gel pictures of PCR products for each genotype are provided in *Figure* S3.

**Measurement of inflammatory cytokines in serum:**

Serum/plasma samples were analyzed for IL-6, TNF-α, GDF-8 (myostatin), IGF-1, and FGF23 by enzyme-linked immunosorbent assay (ELISA) by commercially available kits, (My BioSource International, Camarillo, CA), according to manufacturer’s instructions.

**Supplementary References**

S1. Marzetti E, Leeuwenburgh C. Skeletal muscle apoptosis, sarcopenia and frailty at old age. Exp Gerontol. 2006;41:1234-8.

S2. Dodds RM, Granic A, Robinson SM, Sayer AA. Sarcopenia, long-term conditions, and multimorbidity: findings from UK Biobank participants. J Cachexia Sarcopenia Muscle. 2020;11:62-8.

S3. Shah SV, Rajapurkar MM, Baliga R. The role of catalytic iron in acute kidney injury. Clin J Am Soc Nephrol. 2011;6:2329-31.

S4. Sohal RS, Wennberg-Kirch E, Jaiswal K, Kwong LK, Forster MJ. Effect of age and caloric restriction on bleomycin-chelatable and nonheme iron in different tissues of C57BL/6 mice. Free Radic Biol Med. 1999;27:287-93.

S5. Altun M, Edstrom E, Spooner E, Flores-Moralez A, Bergman E, Tollet-Egnell P, et al. Iron load and redox stress in skeletal muscle of aged rats. Muscle Nerve. 2007;36:223-33.

S6. Xu J, Jia Z, Knutson MD, Leeuwenburgh C. Impaired iron status in aging research. Int J Mol Sci. 2012;13:2368-86.

S7. Haddad F, Zaldivar F, Cooper DM, Adams GR. IL-6-induced skeletal muscle atrophy. J Appl Physiol (1985). 2005;98:911-7.

S8. Doria E, Buonocore D, Focarelli A, Marzatico F. Relationship between human aging muscle and oxidative system pathway. Oxid Med Cell Longev. 2012;2012:830257.

S9. Powers SK, Morton AB, Ahn B, Smuder AJ. Redox control of skeletal muscle atrophy. Free Radic Biol Med. 2016;98:208-17.

S10. Powers SK, Kavazis AN, McClung JM. Oxidative stress and disuse muscle atrophy. J Appl Physiol (1985). 2007;102:2389-97.

S11. Kondo H, Miura M, Nakagaki I, Sasaki S, Itokawa Y. Trace element movement and oxidative stress in skeletal muscle atrophied by immobilization. Am J Physiol. 1992;262:E583-90.

S12. Bose C, Megyesi JK, Shah SV, Hiatt KM, Hall KA, Karaduta O, et al. Evidence Suggesting a Role of Iron in a Mouse Model of Nephrogenic Systemic Fibrosis. PLoS One. 2015;10:e0136563.

S13. Lele SS, Mukhopadhyay BN, Mardikar MM, Patel TA, Vasavada AK, Banker DN, et al. Impact of catalytic iron on mortality in patients with acute coronary syndrome exposed to iodinated radiocontrast-The Iscom Study. Am Heart J. 2013;165:744-51.

S14. Bose C, Kshirsagar S, Vijayan M, Kumar S, Singh SP, Hindle A, et al. The role of RLIP76 in oxidative stress and mitochondrial dysfunction: Evidence based on autopsy brains from Alzheimer's disease patients. Biochim Biophys Acta Mol Basis Dis. 2024;1870:166932.

S15. Saini A, Al-Shanti N, Faulkner SH, Stewart CE. Pro- and anti-apoptotic roles for IGF-I in TNF-alpha-induced apoptosis: a MAP kinase mediated mechanism. Growth Factors. 2008;26:239-53.

S16. Lenk K, Schuler G, Adams V. Skeletal muscle wasting in cachexia and sarcopenia: molecular pathophysiology and impact of exercise training. J Cachexia Sarcopenia Muscle. 2010;1:9-21.

S17. Sheffield-Moore M, Urban RJ. An overview of the endocrinology of skeletal muscle. Trends Endocrinol Metab. 2004;15:110-5.

S18. Padhi D, Higano CS, Shore ND, Sieber P, Rasmussen E, Smith MR. Pharmacological inhibition of myostatin and changes in lean body mass and lower extremity muscle size in patients receiving androgen deprivation therapy for prostate cancer. J Clin Endocrinol Metab. 2014;99:E1967-75.

S19. Loecker C, Schmaderer M, Zimmerman L. Frailty in Young and Middle-Aged Adults: An Integrative Review. J Frailty Aging. 2021;10:327-33.

S20. Siriett V, Platt L, Salerno MS, Ling N, Kambadur R, Sharma M. Prolonged absence of myostatin reduces sarcopenia. J Cell Physiol. 2006;209:866-73.

S21. Liu CM, Yang Z, Liu CW, Wang R, Tien P, Dale R, et al. Myostatin antisense RNA-mediated muscle growth in normal and cancer cachexia mice. Gene Ther. 2008;15:155-60.

S22. Lele S, Shah S, McCullough PA, Rajapurkar M. Serum catalytic iron as a novel biomarker of vascular injury in acute coronary syndromes. EuroIntervention. 2009;5:336-42.

S23. Rajapurkar MM, Shah SV, Lele SS, Hegde UN, Lensing SY, Gohel K, et al. Association of catalytic iron with cardiovascular disease. Am J Cardiol. 2012;109:438-42.

S24. Kell DB. Towards a unifying, systems biology understanding of large-scale cellular death and destruction caused by poorly liganded iron: Parkinson's, Huntington's, Alzheimer's, prions, bactericides, chemical toxicology and others as examples. Arch Toxicol. 2010;84:825-89.

S25. Ke Y, Ming Qian Z. Iron misregulation in the brain: a primary cause of neurodegenerative disorders. Lancet Neurol. 2003;2:246-53.

S26. Cerullo F, Gambassi G, Cesari M. Rationale for antioxidant supplementation in sarcopenia. J Aging Res. 2012;2012:316943.

S27. Zanders L, Kny M, Hahn A, Schmidt S, Wundersitz S, Todiras M, et al. Sepsis induces interleukin 6, gp130/JAK2/STAT3, and muscle wasting. J Cachexia Sarcopenia Muscle. 2022;13:713-27.
